# PTBP1 enforces ATR-CHK1 signaling determining the potency of CDC7 inhibitors

**DOI:** 10.1016/j.isci.2023.106951

**Published:** 2023-05-26

**Authors:** Anja Göder, Aisling Quinlan, Michael D. Rainey, Declan Bennett, Daniel Shamavu, Jacqueline Corso, Corrado Santocanale

**Affiliations:** 1 Centre for Chromosome Biology, School of Biological and Chemical Sciences, University of Galway, Galway H91W2TY, Ireland; 2 School of Mathematical & Statistical Sciences, University of Galway, Galway H91TK33, Ireland

**Keywords:** Biological sciences, Molecular genetics, Molecular biology

## Abstract

CDC7 kinase is crucial for DNA replication initiation and fork processing. CDC7 inhibition mildly activates the ATR pathway, which further limits origin firing; however, to date the relationship between CDC7 and ATR remains controversial. We show that CDC7 and ATR inhibitors are either synergistic or antagonistic depending on the degree of inhibition of each individual kinase. We find that Polypyrimidine Tract Binding Protein 1 (PTBP1) is important for ATR activity in response to CDC7 inhibition and genotoxic agents. Compromised PTBP1 expression makes cells defective in RPA recruitment, genomically unstable, and resistant to CDC7 inhibitors. PTBP1 deficiency affects the expression and splicing of many genes indicating a multifactorial impact on drug response. We find that an exon skipping event in RAD51AP1 contributes to checkpoint deficiency in PTBP1-deficient cells. These results identify PTBP1 as a key factor in replication stress response and define how ATR activity modulates the activity of CDC7 inhibitors.

## Highlights

• ATR activity determines the potency of CDC7 inhibitors
• PTBP1 loss of function impairs the replication stress response and ATR activation
• Repression of replication origin firing is compromised in PTBP1-deficient cells
• Correct splicing of RAD51AP1 requires PTBP1 and is important for ATR signaling

## Graphical abstract

**Figure:**
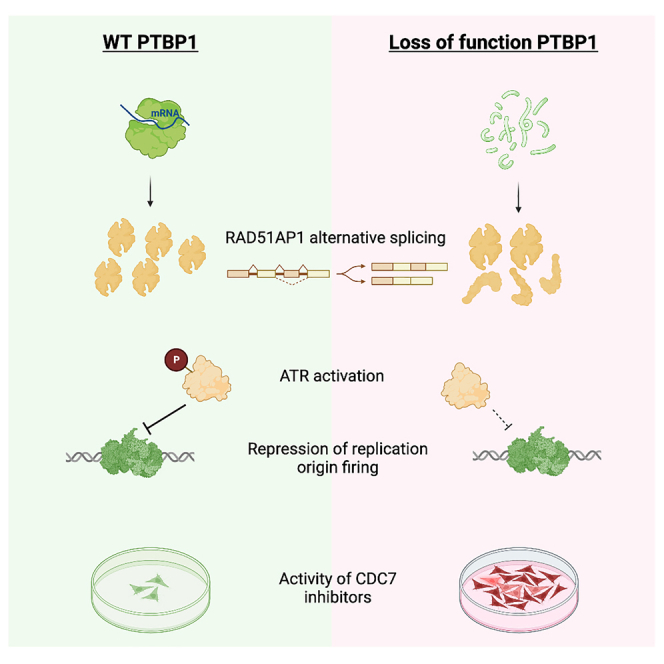

## Introduction

The replication of genomic DNA is highly regulated both at the level of initiation, which occurs in a coordinated manner from multiple origins of replication, as well as at each individual replication fork, which can encounter obstacles leading to replication fork stalling and collapse.^1^^,^^2^^,^^3^^,^^4^ Defining the mechanisms that coordinate replication initiation and elongation is an important task to understand how genome stability is maintained during DNA replication and it can also have therapeutic implications for cancer therapy.

Two kinases, CDC7 and ATR, play essential roles in this process with both being involved in controlling initiation and elongation.^5^^,^^6^^,^^7^^,^^8^^,^^9^^,^^10^^,^^11^ In initiation, CDC7 phosphorylates several subunits of the MCM helicase allowing for the formation of the CDC45-MCM-GINS (CMG) complex and origin activation.^6^^,^^11^^,^^12^ CDC7-dependent phosphorylation of the MCM complex is counteracted by RIF1-PP1 phosphatase; thus, the efficiency of initiation is dictated by a balance between phosphatase and kinase activity.^13^^,^^14^ ATR was also reported to phosphorylate the MCM complex and in yeast it can act as a priming event for subsequent CDC7 phosphorylation suggesting a role in promoting origin firing.^9^^,^^15^ However, multiple studies have since shown that ATR mainly acts as a negative regulator of origin firing and in human cells it does so by downregulating CDK1 activity, thus reinforcing the RIF1/PP1 axes, both during unperturbed S-phase and in response to genotoxic agents.^5^^,^^16^^,^^17^

During elongation, CDC7 is involved in DNA damage tolerance pathways, like translesion synthesis (TLS), in yeast as well as human cells.^18^ More recently, we showed that human CDC7 kinase physically associates with ongoing and stalled replication forks, where it promotes fork processing and restart in a MRE11-dependent manner. MRE11, together with EXO1 and other nucleases, generate extended ssDNA in the proximity of the fork resulting in RPA accumulation and activation of ATR kinase.^19^^,^^20^ ATR is then responsible for the phosphorylation of many factors, which help in preserving fork integrity and arresting the cell cycle, thus limiting DNA damage and genome instability.^16^^,^^21^

Both ATR and CDC7 inhibitors (ATRis and CDC7is) show excellent anti-tumor activity in a variety of preclinical cancer models and are currently being explored as potential anti-cancer agents in clinical trials.^22^^,^^23^^,^^24^^,^^25^

Among the CDC7is, XL413 and TAK-931 have a good selectivity profile and a different mechanism of action compared to earlier generations of CDC7 inhibitors.^26^ Extensive preclinical studies performed with TAK-931 have suggested that tumors experiencing high levels of replication stress are more sensitive to CDC7 inhibition.^27^ This raises the expectation that the combination of CDC7is and checkpoint inhibitors, including ATRis, CHK1 inhibitors (CHK1is), and WEE1 inhibitors (WEE1is), would be an attainable and effective strategy by increasing underlying replication stress of cancer cells.

Nevertheless, the relationship between CDC7 and the ATR pathway is not well understood, with reports indicating that CDC7is and checkpoint inhibitors have additive or synergistic effects in cell killing, whereas others indicate antagonism.^10^^,^^27^ Furthermore, mechanistically, it is highly debated whether CDC7 should be considered as an effector or a direct target of the ATR pathway.^28^^,^^29^^,^^30^^,^^31^ Such discrepancies particularly affect the rational clinical development of CDC7is and to a lesser extent of ATRis.

With the aim of identifying genes, which contribute to the antiproliferative activity of CDC7is, we conducted a genome-wide CRISPR/Cas9 KO screen with cells that had been treated with high doses of the CDC7i XL413. In that study, we identified ETAA1 as the relevant subunit activating ATR, restraining DNA synthesis and proliferation in response to CDC7 inhibition. However, we also reported that co-inhibition of CDC7 and ATR leads to cell death through mitotic catastrophe,^32^ an observation that has since been recapitulated by others.^33^ Such apparent contradiction likely indicates an ambiguous relationship between the two kinase activities and that the phenotypes observed may strongly depend on the levels of inhibition of the two kinases, achieved by either genetic or pharmacological means.

Here we reveal the role of Polypyrimidine tract binding protein 1 (PTBP1), which was identified in the aforementioned CRISPR screen, as a novel modulator of the ATR pathway. PTBP1’s main function, as a member of the heterogeneous nuclear ribonucleoprotein (hnRNP) subfamily, is in RNA metabolism: controlling mRNA alternative splicing, polyadenylation, translocation, and stability.^34^^,^^35^^,^^36^^,^^37^^,^^38^ It has also been shown to influence the initiation of translation through internal ribosome entry site-mediated translation.^39^ It has four tandem RNA recognition motifs (RRM) and an N-terminal nuclear localization signal (NLS).^40^ PTBP1 interacts with mRNA at polypyrimidine-rich regions, namely UCUU-rich sequences in exonic and flanking intronic areas^39^ and it usually promotes the inclusion of the exon.^41^^,^^42^ Because of its biochemical activity, PTBP1 has a pleiotropic role in cells and has been implicated in many biological processes, from the regulation of the pro-inflammatory senescence-associated secretory phenotype (SASP) and B-cell maturation to cancer cell proliferation and invasion.^43^^,^^44^^,^^45^^,^^46^ Intriguingly, chemo-genetic screens have suggested that PTBP1 may affect cell viability to some genotoxic agents in RPE1 cells.^47^ However, the exact involvement of PTBP1 in the response to these agents has not been studied yet.

We now show that PTBP1 loss of function causes partial resistance to CDC7 inhibition by reducing ATR activation in response to CDC7is as well as DNA damaging agents. We also show that low levels of ATR inhibition, similar to PTBP1 loss of function, allows more efficient DNA synthesis in the presence of CDC7is. Impaired ATR activation in PTBP1-deficient cells correlates with deficient RPA chromatin binding and focal recruitment suggesting altered frequency of fork stalling or processing. Finally, we identify RAD51AP1, which is defectively spliced and expressed in PTBP1-deficient cells, as an important component of the replication stress response.

## Results

**Figure 1:**
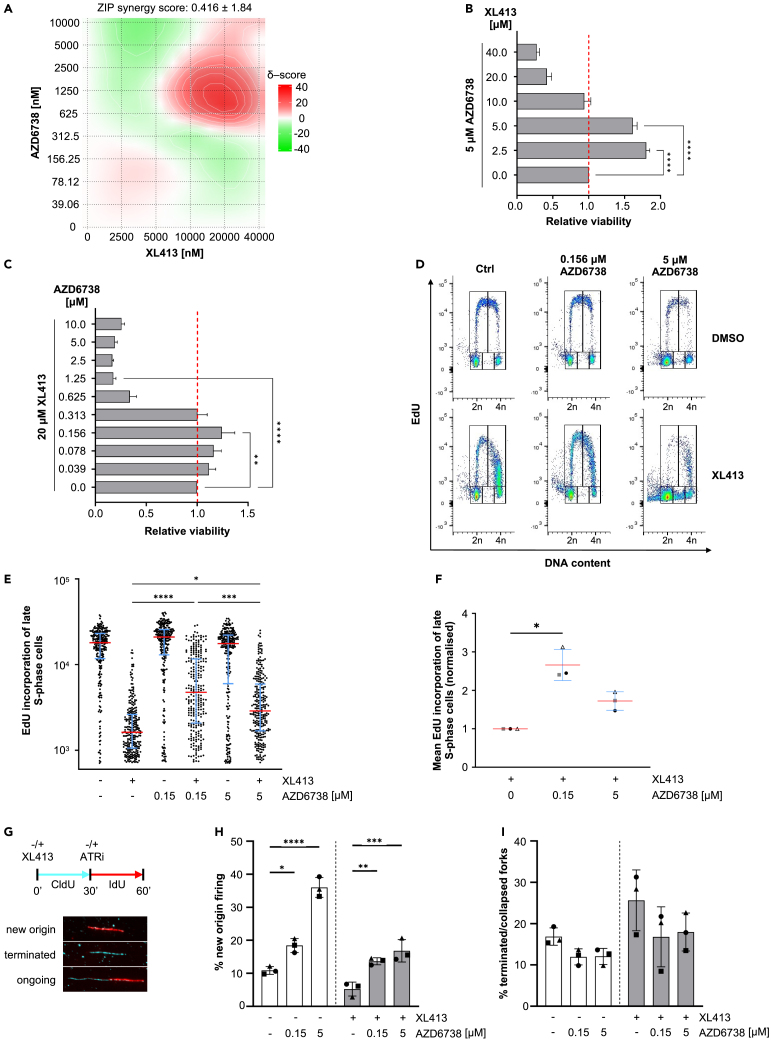
Partial inhibition of ATR confers resistance to CDC7is while full inhibition results in sensitisation (A) MCF10A cells were treated with indicated doses of XL413 and AZD6738 for 72 h followed by resazurin assay to analyze cell viability. 2D synergy map was generated using normalized viability scores of three independent resazurin assays. Antagonistic (green, δ score <−10), additive (δ score −10 to 10) or synergistic (red, δ score >10) dose regions are highlighted. (B) Data described in A were further analyzed. Viability was determined relative to samples treated with 5 μM AZD6728 (dotted line). Data represent three independent experiments performed in technical triplicates, mean ± SD. Statistical analysis was performed using one-way ANOVA and Dunnett’s multiple comparison test (∗∗∗∗p < 0.0001). (C) Additional analysis of data presented in A. Viability was determined relative to samples treated with 20 μM XL413 (dotted line). Data represent three independent experiments performed in technical triplicates, mean ± SD. Statistical analysis was performed using one-way ANOVA and Dunnett’s multiple comparison test (∗∗p < 0.01; ∗∗∗∗p < 0.0001). (D) MCF10A EditR cells were co-treated with 20 μM XL413, DMSO and/or indicated doses of AZD6738 for 24 h. For flow cytometry analysis, cells were labeled with 10 μM EdU for 30 min before harvesting. Representative images from one of three independent experiments are shown. (E) Fluorescence intensity, proportional to EdU incorporation, in late S-phase cells for representative experiment described in D. Red lines indicate the medians and blue lines show the interquartile range extending from the 25^th^ to the 75^th^ percentile for 266 cells. Statistical analysis was performed using one-way ANOVA, with Tukey’s multiple comparison test (∗p < 0.05; ∗∗∗p < 0.001; ∗∗∗∗p < 0.0001). (F) Mean fluorescence intensity in late S-phase cells of three independent experiments expressed as a ratio relative to 20 μM XL413-treated samples. Red and blue lines show the means ± SDs. Statistical analysis was performed using Kruskal-Wallis ANOVA, with Dunn’s multiple comparison test (∗p < 0.05). (G) MCF10A EditR cells were either treated with DMSO or 20 μM XL413 and labeled with CldU (cyan) for 30 min. CldU was washed off, AZD6738 (0.156 μM or 5 μM, as indicated) or DMSO were added and cells were labeled with IdU for 30 min in the continued presence or absence of XL413. Representative images of scored fibers are shown. See also Figure S1. (H) Percentages of IdU only tracks (new origin firing) are plotted for the DNA fiber assay described in G. At least 150 replication tracks were analyzed for each condition of three independent experiments. Data is presented as mean ± SD. Statistical analysis was performed using one-way ANOVA, with Tukey’s multiple comparison test (∗p < 0.05; ∗∗p < 0.01; ∗∗∗p < 0.001; ∗∗∗∗p < 0.0001). (I) Percentages of CldU only tracks (terminated/collapsed forks) are plotted for the DNA fiber assay described in G. At least 150 replication tracks were analyzed for each condition of three independent experiments. Data is presented as mean ± SD.

We previously reported that in MCF10A cells CDC7 inhibition can cause cell growth arrest, replication stress and ATR activation.^32^ To better understand how the antiproliferative activity of CDC7is may be affected by ATR, we treated MCF10A cells with a well characterized CDC7i (XL413) and an ATRi (AZD6738) at increasing concentrations either alone or in combination. Cell viability was assessed after three days using a resazurin assay. We discovered that at most doses ATRi and CDC7i have an additive (δ score: −10 to 10) or synergistic (δ score: >10) effect in preventing proliferation, possibly leading to cell death with a maximum δ score of 26.73 (Figure 1A). However, low doses of XL413 suppressed AZD6738-induced loss of viability (Figures 1A and 1B), while low doses of AZD6738 allowed cells to grow better in presence of high doses of XL413 (Figures 1A and 1C). More specifically, we noticed that 0.156 μM AZD6738, allows MCF10A cells to better proliferate in the presence of 20 μM XL413, whereas doses of ≥0.625 μM AZD6738 drastically decreased the number of living cells (Figure 1C). Using a flow cytometry-based assay, we observed that while, on its own, 20 μM XL413 drastically reduced DNA synthesis, causing cells to accumulate in mid-to-late S-phase of the cell cycle, the addition of 0.156 μM AZD6738 allows DNA synthesis to proceed more efficiently and cells distribute more normally throughout the phases of the cell cycle. In contrast, cells treated with high doses of both compounds dispersed symmetrically around G1 DNA content with many cells not incorporating further EdU into their DNA (Figures 1D–1F), a phenotype that we previously correlated with mitotic catastrophe.^32^ DNA fiber assay was then performed to provide a more in-depth analysis of the replication dynamics of cells treated with CDC7is and ATRis. MCF10A EditR cells were treated with 20 μM XL413 or DMSO and labeled with CldU for 30 min followed by the addition of either 0.156 μM or 5 μM AZD6738 and labeling with IdU in the continued presence or absence of XL413 (Figure 1G). We found that the addition of AZD6738, at both low and high doses, significantly increased origin firing both in the presence or absence of CDC7i whereas we did not observe a statistically significant change in the number of terminating forks (Figures 1G–1I and S1).

These results indicate that the efficiency of CDC7is in limiting DNA synthesis and restraining proliferation requires robust ATR activity, but a severely compromised ATR response can eliminate cells exposed to CDC7is.

### PTBP1 deficiency suppresses the antiproliferative activity of CDC7 inhibitors

In a previously published CRISPR/Cas9 KO screen, we identified several genes that when edited may confer resistance to XL413, among which were RIF1, ETAA1, and PTBP1. We showed that ETAA1 depletion hinders the ATR response triggered by CDC7is allowing more origin firing and more efficient DNA synthesis to occur, similarly to RIF1 depletion and low levels of ATR inhibition by AZD6738 (ref. 32 and Figure 1).

To validate that loss of PTBP1 provided cells with a proliferative advantage when CDC7 is inhibited, a 16-day competitive proliferation assay was performed. MCF10A EditR cells, stably expressing Cas9, were transduced with a lentiviral vector that co-expressed GFP and one of two short guide RNAs (sgRNAs) targeting *PTBP1*, sgRNA4 or sgRNA6, or alternatively with an empty vector (EV) control (Figure S2A). Starting with a mixed population of 5–10% GFP-positive cells, we assessed the percentage of GFP-positive cells following a treatment with 20 μM XL413 for 16 days. Relative to the EV control, sgRNA4 and sgRNA6 transfected cells showed an increase in the percentage of GFP-positive cells in the population (Figure S2A). This indicates that editing the *PTBP1* gene confers a proliferative advantage on CDC7 inhibition.

From the pools of transduced cells, we isolated two independent clones, one for each sgRNA, with mutations in the *PTBP1* gene (Figure S2B). Surprisingly, we found that neither cell line harbored a null mutation in the *PTBP1* gene: clone 4.1 had a 3-nucleotide homozygous deletion in exon 5 resulting in the loss of Ile99 within the RRM1 leading to protein instability, as PTBP1 protein was barely detectable by western blotting and immunofluorescence microscopy (Figures S2B–S2E). In contrast, clone 6.6 carried a wild type allele and an allele with a 9-nucleotide loss leading to an in-frame deletion of three amino acids within the RRM2 in heterozygosis (Figure S2B). This small in-frame deletion likely also leads to protein instability as the level of PTBP1 protein was decreased compared to the parental MCF10A EditR cells (Figures S2C and S2D).

**Figure 2:**
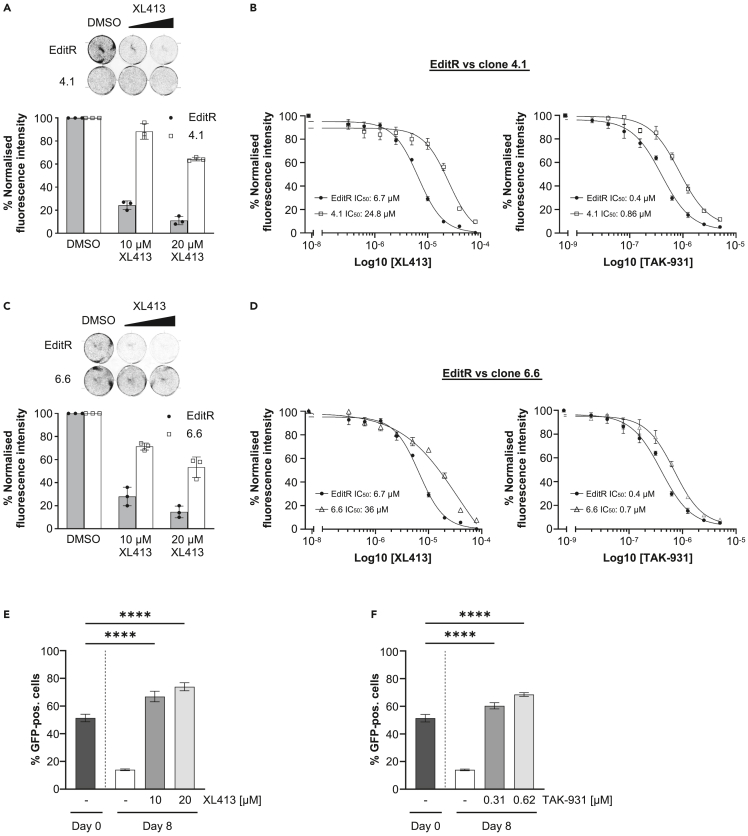
PTBP1 deficiency supresses the antiproliferative effect of CDC7 inhibitors (A) MCF10A EditR and PTBP1 mutant clone 4.1 cells were treated with DMSO, 10 μM or 20 μM XL413 and incubated for 72 h. Cells were stained with crystal violet and intensity of the stain was measured. Representative image of the staining is displayed alongside graphs depicting the relative intensity of the crystal violet stain compared to DMSO controls. Data represent three biological repeats, mean ± SD. See also Figure S2. (B) MCF10A EditR and PTBP1 mutant clone 4.1 cells were treated with 0.3125 to 80 μM XL413 (left) or 0.0195 to 5 μM TAK-931 (right) and incubated for 72 h. Cells were stained with resazurin and fluorescence intensity was measured. Graphs show growth inhibition curves and IC_50_ values. Data represents three biological repeats performed in technical triplicates, mean ± SD. (C) MCF10A EditR and PTBP1 clone 6.6 cells were treated and analyzed as described in A. Data represent three biological repeats, mean ± SD. (D) MCF10A EditR and PTBP1 mutant clone 6.6 cells were treated and analyzed as described in B. Data represents three biological repeats performed in technical triplicates, mean ± S.D. (E) MCF10A EditR (GFP-negative) and PTBP1 mutant clone 4.1 cells (GFP-positive) were co-cultured in the same plate and treated with DMSO or indicated concentrations of XL413 for 8 days. Percentage of GFP-positive cells was assessed by flow cytometry at Day 0 and Day 8. Data represent four biological repeats, mean ± SD. Statistical analysis was performed using one-way ANOVA and Dunnett’s multiple comparison test (∗∗∗∗p < 0.0001). (F) MCF10A EditR (GFP-negative) and PTBP1 mutant clone 4.1 cells (GFP-positive) were co-cultured in the same plate and were treated with DMSO or indicated concentration of TAK-931 for 8 days. Analysis was performed as described in E. Data represent four biological repeats, mean ± SD.

To further confirm, that PTBP1 is required to restrain proliferation in cells treated with CDC7is, MCF10A EditR cells and PTBP1 mutant clones 4.1 and 6.6 were treated with DMSO, 10 μM or 20 μM XL413 for 72 h. Cells were then stained with crystal violet to assess cell viability. The intensity of crystal violet stain was quantified for each treatment and normalized to its DMSO control. Treatment with XL413 restricted cell proliferation in the EditR cells and to a lesser extent in clones 4.1 and 6.6; notably the extent to which the antiproliferative effect of XL413 was suppressed correlated with the residual amount of PTBP1 protein in these cells (Figures 2A and 2C). Dose response experiments with two independent CDC7is, XL413 and TAK-931, further demonstrated that PTBP1 mutant clones 4.1 and 6.6 are more resistant to CDC7 inhibition showing a clear shift in both XL413 and TAK-931 growth inhibition curves and IC_50_ compared to the parental cell line (Figures 2B and 2D).

To confirm that the proliferative advantage of the PTBP1 mutant clones persists even after prolonged treatment with CDC7is, we extended the treatment to eight consecutive days in a competitive proliferation assay with MCF10A EditR cells. Equal numbers of MCF10A EditR and GFP-expressing PTBP1 mutant clone 4.1 (Figure S2E) were mixed and cultured in DMSO or increasing doses of XL413 and TAK-931 over eight days before assessing the percentage of GFP-positive cells. In absence of CDC7is we observed that MCF10A EditR cells were accumulating in the population, but importantly the treatment with either one of the CDC7is resulted in a significant increase in the percentage of GFP-expressing cells, indicating that PTBP1 mutant clone 4.1 is outcompeting MCF10A EditR cells only in the presence of CDC7is (Figures 2E and 2F).

**Figure 3:**
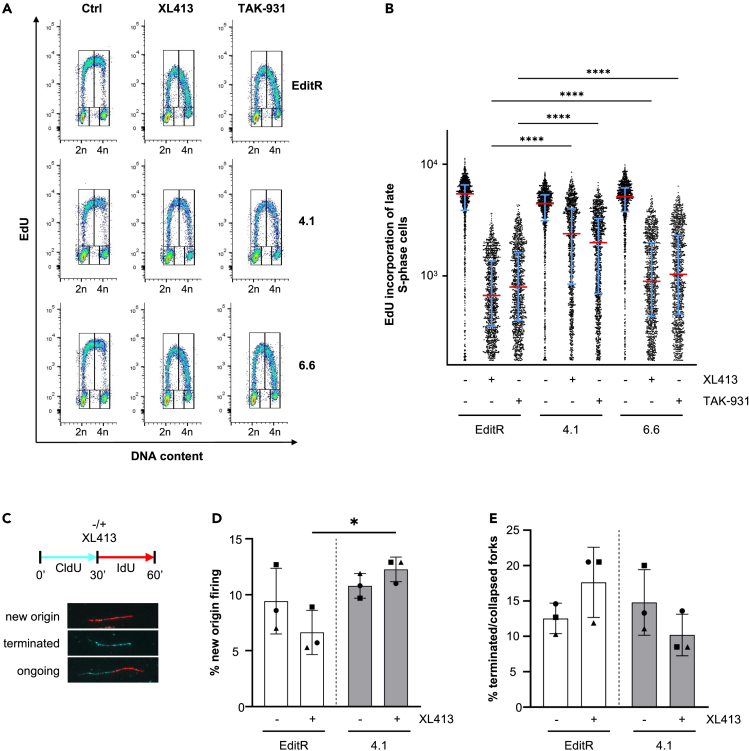
PTBP1 contributes to the inhibition of DNA synthesis caused by CDC7 inhibition (A) MCF10A EditR, PTBP1 mutant clone 4.1 or 6.6 cells were treated with DMSO, 20 μM XL413 or 0.625 μM TAK-931 for 24 h. For flow cytometry analysis, cells were labeled with 10 μM EdU 30 min before harvest. Representative images from one of three independent experiments are shown. (B) Fluorescence intensity, proportional to EdU incorporation, in late S-phase cells for representative experiment displayed in A. Red lines indicate the medians and blue lines show the interquartile range extending from the 25^th^ to the 75^th^ percentile of 1085 cells. Statistical analysis was performed using one-way ANOVA, with Tukey’s multiple comparison test (∗∗∗∗p < 0.0001). (C) MCF10A EditR or PTBP1 mutant clone 4.1 cells were labeled with CldU (cyan) for 30 min. CldU was washed off and cells were labeled with IdU for 30 min in the presence of 20 μM XL413 or DMSO. Representative images of scored fibers are shown. See also Figure S3. (D) Percentages of IdU only tracks indicating new origin firing in the experiment described in C. At least 250 tracks were analyzed for each condition of three independent experiments. Data is presented as mean ± SD. Statistical analysis was performed using one-way ANOVA, with Tukey’s multiple comparison test (∗p < 0.05). (E) Percentages of CldU only tracks, indicating terminated/collapsed forks in the experiment described in C. At least 250 replication tracks were analyzed for each condition of three independent experiments. Data is presented as mean ± SD.

To understand if the resistance to CDC7is in PTBP1 mutant cells was related to DNA replication, MCF10A EditR and clones 4.1 and 6.6 cells were treated with either XL413 or TAK-931 for 24 h and DNA synthesis as well as cell cycle distribution was analyzed by flow cytometry. In parental cells CDC7i treatment led to the characteristic accumulation of cells in late S-phase with a lower rate of DNA synthesis, however PTBP1 mutant clone 4.1, and to a lesser extent clone 6.6, displayed near normal levels of DNA synthesis despite the presence of CDC7is (Figures 3A and 3B). Similarly, PTBP1 depletion by siRNA increased the rate of DNA synthesis and reduced the accumulation of cells in late S-phase upon CDC7 inhibition (Figures S3A–S3D).

As shown for the combination of ATRis and CDC7is, unscheduled origin firing can partially overcome the effects of CDC7is on the rate of DNA synthesis (Figures 1D–1I). To investigate whether PTBP1 deficiency reduces the requirement for CDC7 activity for origin activation, MCF10A EditR and PTBP1 mutant clone 4.1 cells were labeled with CldU for 30 min, then treated with 20 μM XL413 or DMSO and labeled with IdU. Analysis of replication products indicated that XL413 treatment, while inhibiting origin firing and inducing low levels of fork stalling in MCF10A EditR had no significant effect in PTBP1 mutant clone 4.1 (Figures 3C–3E and S3E-S3G).

All together, these data provide compelling evidence that PTBP1 contributes to restricting DNA synthesis and origin firing, thus delaying S-phase progression when CDC7 is inhibited. As the magnitude of this effect is dependent on the residual amount of functional PTBP1, all further experiments were performed with clone 4.1.

### ATR signaling is partially impaired in PTBP1-deficient cells

**Figure 4:**
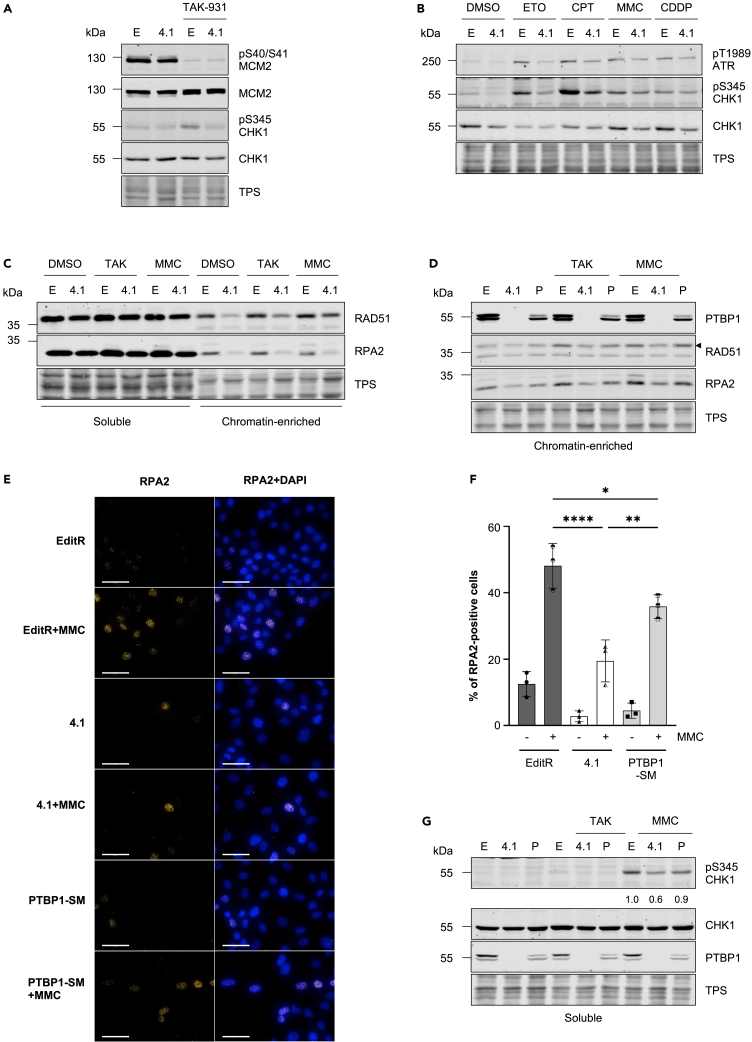
PTBP1 deficiency leads to partial loss of ATR-dependent checkpoint signaling (A) MCF10A EditR (E) or PTBP1 mutant clone 4.1 (4.1) cells were treated with 0.625 μM TAK-931 for 24 h. Whole cell extracts were prepared and analyzed by immunoblotting with indicated antibodies. Total protein staining (TPS) was used as loading control. Data are representative of three independent experiments. (B) MCF10A EditR (E) or PTBP1 mutant clone 4.1 (4.1) cells were treated with 10 μM etoposide (ETO), 50 nM camptothecin (CPT), 200 nM mitomycin C (MMC) or 5 μM *cis*-platin (CDDP) for 24 h. Whole cell extracts were prepared and samples were analyzed with indicated antibodies via immunoblotting. TPS was used as loading control. Data are representative of two independent experiments. (C) MCF10A EditR (E) or PTBP1 mutant clone 4.1 (4.1) cells were treated with DMSO, 0.625 μM TAK-931 or 200 nM MMC for 24 h. Soluble and chromatin-enriched fractions were prepared and analyzed by immunoblotting with indicated antibodies. TPS was used as loading control. Data are representative of three independent experiments. (D) MCF10A EditR (E), PTBP1 mutant clone 4.1 (4.1) and clone 4.1 expressing PTBP1-SM (P) cells were treated with DMSO, 0.625 μM TAK-931 or 200 nM MMC for 24 h. Chromatin-enriched fractions were prepared and analyzed by immunoblotting with indicated antibodies. TPS was used as loading control. Triangle marks RAD51 band. Data are representative of three independent experiments. See also Figure S4. (E) MCF10A EditR, clone 4.1 and clone 4.1 expressing PTBP1-SM cells were treated with 200 nM MMC for 24 h before fixation. RPA2 (yellow) was analyzed by immunofluorescence. DAPI was used to visualize nuclei (blue). Representative images of three independent experiments are shown (Scale bar, 50 μm). Brightness of images was adjusted (across all samples) to aid visualization. (F) Quantification of RPA2 foci per nucleus was performed by automated analysis with the Operetta high-throughput microscope and Harmony analysis software. Cells were classified as “RPA2-positive” if more than 10 foci were detected. Graphs represent three biological repeats and at least 150 cells were analyzed per condition and experiment, mean ± SD. Statistical analysis was performed using one-way ANOVA with Tukey’s multiple comparison test (∗p < 0.05; ∗∗p < 0.01; ∗∗∗∗p < 0.0001). (G) MCF10A EditR (E), PTBP1 mutant clone 4.1 (4.1) and clone 4.1 expressing PTBP1-SM (P) cells were treated with DMSO, 0.625 μM TAK-931 or 200 nM MMC for 24 h. Soluble fractions were prepared and analyzed by immunoblotting with indicated antibodies. TPS was used as loading control. Numbers represent quantification of pS345 CHK1 signal normalized to TPS and relative to EditR cells treated with MMC. Blots are representative of three independent experiments.

Our analysis indicates that PTBP1 deficiency phenocopies the partial inhibition of ATR by AZD6738 suggesting that in PTBP1 mutant clone 4.1 the ATR response may be impaired. To test this hypothesis, we treated MCF10A EditR and clone 4.1 cells with either CDC7i or several drugs causing replication stress and DNA damage and assessed the activation of ATR signaling by semiquantitative western blotting. This analysis revealed that CHK1-S345 phosphorylation, a downstream marker of ATR activity, was reduced in PTBP1-deficient cells on TAK-931, etoposide, camptothecin, mitomycin C (MMC) and *cis*-platin treatment (Figures 4A and 4B) and that, when analyzed, ATR-T1989 autophosphorylation was also reduced (Figure 4B).

To understand the reasons for the lack of checkpoint activation, extracts prepared from cells either mock-treated or treated with TAK-931 and MMC were fractionated into soluble and chromatin-enriched fraction and analyzed by western blotting. Notably, we observed that the levels of RAD51 and RPA associated with chromatin were decreased in PTBP1-deficient cells (Figures 4C and S4C). RPA foci formation in MMC treated cells was also defective (Figures 4E and 4F)

To further prove that PTBP1 was indeed responsible for these phenotypes, clone 4.1 cells were transduced with lentiviruses expressing a *PTBP1* allele carrying a silent mutation in the Cas9 PAM site, thus making it resistant to Cas9 mediated cleavage (PTBP1-SM) and consequently generating a polyclonal population of cells expressing a functional PTBP1 protein. Western blotting and immunofluorescence microscopy analysis indicated that PTBP1-SM did not fully rescue the normal levels of PTBP1 expression, likely because of the heterogeneous expression in the polyclonal population (Figures 4D, S4A, and S4B). However, it was sufficient to mostly restore MMC-induced RAD51 and RPA chromatin association (Figures 4D and S4C), MMC-induced RPA focal formation (Figures 4E and 4F), and CHK1-S345 phosphorylation (Figure 4G).

Thus, PTBP1 determines the amount of RPA-coated ssDNA that is generated at replication forks, both during normal and challenged DNA replication, which in turn limits the extent of ATR activation.

**Figure 5:**
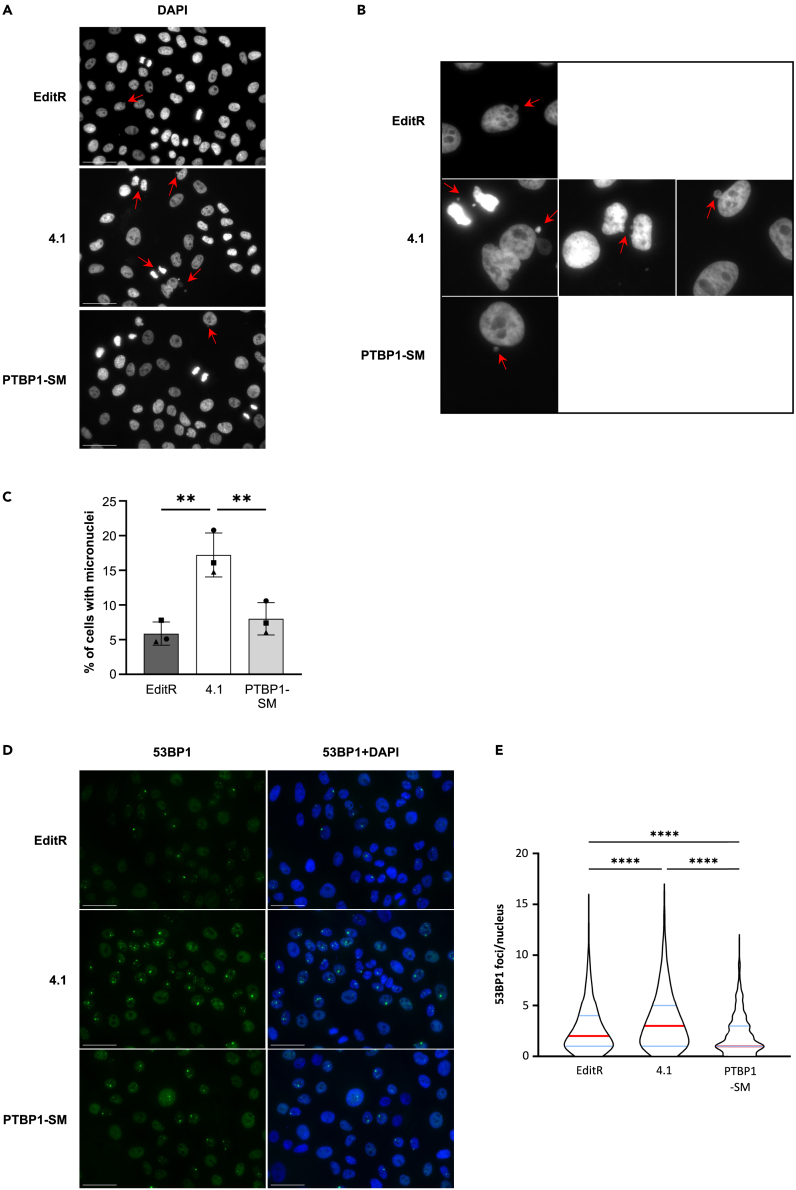
PTBP1 deficiency leads to genomic instability and induction of 53BP1 nuclear bodies in G1 cells (A) MCF10A EditR, PTBP1 mutant clone 4.1 and clone 4.1 expressing PTBP1-SM (PTBP1-SM) cells were fixed, stained with DAPI to visualize DNA, and analyzed by fluorescence microscopy. Representative images of three independent experiments are shown (Scale bar, 50 μm). Red arrows indicate micronuclei. Brightness of images was adjusted for all samples to aid visualization. See also Figure S5. (B) Cells with micronuclei (red arrows) in representative images (A) were enlarged to aid visualization. (C) Graph shows percentage of cells with micronuclei for three independent experiments, mean ± SD. At least 330 cells were analyzed per condition for each experiment. Statistical analysis was performed using one-way ANOVA with Tukey’s multiple comparison test (∗∗p < 0.01). (D) MCF10A EditR, PTBP1 mutant clone 4.1 and clone 4.1 expressing PTBP1-SM (PTBP1-SM) cells were fixed, immunostained with anti-53BP1 antibodies (green) and analyzed by immunofluorescence. DAPI was used to visualize DNA (blue). Representative images of three independent experiments are shown (Scale bar, 50 μm. Brightness of images was adjusted for all samples to aid visualization. (E) Number of high intensity, nuclear 53BP1 foci in cells with low DAPI intensity (G1 cells) was determined using ImageJ software. Data are presented as median (red line) ± interquartile range (blue lines). 300 cells were analyzed per sample for three independent experiments. Statistical analysis was performed using one-way ANOVA with Tukey’s multiple comparison test (∗∗∗∗p < 0.0001).

Consistent with poor ATR signaling and problems in DNA replication, many micronuclei were observed in PTBP1 mutant clone 4.1 cells, and their number decreased to almost normal levels on ectopic expression of PTBP1-SM (Figures 5A–5C). More interestingly, PTBP1 mutant clone 4.1 cells displayed a significant increase in large 53BP1 foci in G1 cells compared to either parental or PTBP1-SM cells (Figures 5D and 5E). Such large foci are also known as 53BP1 bodies and have been associated with repair of unreplicated DNA from the previous cell cycle.^48^^,^^49^ Since micronuclei can also be generated by defective mitosis independently from DNA replication, we scored approximately 600 mitotic cells in MCF10A EditR, PTBP1 mutant clone 4.1 and PTBP1-SM cells. Chromatin bridges and lagging chromosomes, were detected in PTBP1-deficient clone 4.1 cells and their number were increased compared to parental and PTBP1-SM cells (Figure S5), although the effect did not reach statistical significance. Altogether, these data indicate that PTBP1 function is required to maintain the normal control of DNA replication and its impairment can lead to genome instability.

### PTBP1 deficiency severely alters the transcriptome

**Figure 6:**
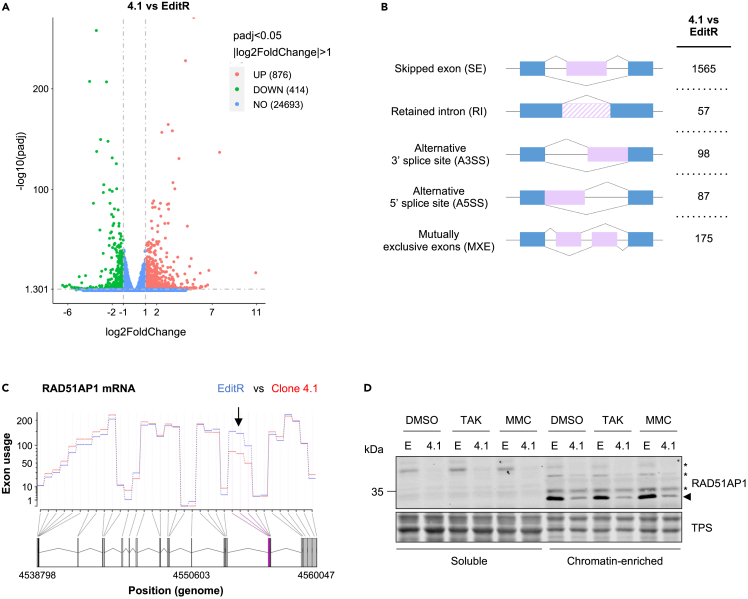
PTBP1 deficiency severely alters the transcriptome in MCF10A cells (A) RNA sequencing was performed in MCF10A EditR and PTBP1 mutant clone 4.1 cells. Volcano plot indicates genes significantly upregulated (red) or downregulated (green) in clone 4.1 versus EditR cells. Threshold was set with adjusted p value < 0.05 and log2 fold change >1. Data represent three independent experiments. See also Figure S6 and Tables S1, S2, and S3. (B) Overview of analyzed types of alternative splicing and number of alternatively spliced transcripts occurring in clone 4.1 vs. EditR cells. (C) DEXSeq plot indicating the exon usage of RAD51AP1 from read counts in clone 4.1 cells (red) and EditR cells (blue). Exon 8, which displays a prominent exon skipping event (arrow) in clone 4.1 cells, is marked in purple. (D) MCF10A EditR (E) and PTBP1 mutant clone 4.1 (4.1) were treated with DMSO, 0.625 μM TAK-931 or 200 nM MMC for 24 h. Soluble and chromatin-enriched fractions were prepared and analyzed by immunoblotting with indicated antibodies. Total protein staining (TPS) was used as loading control. Triangle marks RAD51AP1 band, asterisks indicate potentially modified forms of RAD51AP1. Blot is representative of three independent experiments.

As the canonical function of PTBP1 is in mRNA processing, we hypothesized that the disrupted DNA replication stress response in PTBP1 mutant cells might be due to the differential gene expression and/or alternative splicing in one or more of the genes regulating these processes. Thus, we performed an RNA-seq experiment to compare the transcriptomes of PTBP1 mutant cells and EditR cells. The analysis of differentially expressed genes identified 876 genes significantly upregulated and 414 significantly downregulated in PTBP1-deficient cells compared to the parental cells (Figure 6A and Table S1). Kyoto Encyclopedia of Genes and Genomes (KEGG) and Gene Ontology (GO) analysis indicated that PTBP1 disfunction can affect multiple pathways and processes among which were complement and coagulation cascades, cell adhesion as well as Ras signaling and cytoskeletal regulation, but, to our surprise, the terms and pathways related to DNA replication, DNA repair or cell cycle regulation were not highlighted (Figure S6). Such widespread alteration in gene expression was also matched by many statistically significant alterations in exon usage across numerous genes with skipped exons being the most frequent event occurring in the PTBP1-deficient cells (Figure 6B).

To determine how PTBP1 mutation could possibly confer resistance to CDC7is, we used the GO database^50^^,^^51^ and generated a list of 1243 genes associated with the terms of DNA replication, DNA damage repair, S-phase checkpoint signaling, S-phase progression, and G1-S-phase transition (Table S2). These genes were then compared to the ones either differentially expressed or alternatively spliced in PTBP1 mutant clone 4.1 compared to EditR cells. We found that some of these genes were partially deregulated in PTBP1-deficient cells, but in most cases the differences in the level of expression were limited (Table S1).

Intriguingly, we observed an imbalance in the expression of subunits of several protein complexes, such as the MRN complex where *MRE11* was downregulated by approximately 30%, whereas *RAD50* expression was increased by 40%. In the fork protection complex *CLASPIN* was upregulated 2-fold, whereas *TIMELESS* was downregulated approximately 20%. Similarly, although *ATR* was downregulated by 25%, *ETAA1* was instead upregulated (Table S3). Thus, even though small changes in the expression of single specific genes are unlikely to be solely responsible in determining a strong phenotype, the imbalance in the expression of proteins forming functional complexes may lead to a more accentuated disruption of their function.

When we examined the genes that were alternatively spliced, we observed that in most cases, although statistically significant, such alternate splicing events either occurred at a low frequency or in exons which are seldom utilized, and were thus unlikely to be responsible for the phenotypes described above. However, we found one exception, a prevalent exon skipping event affecting exon 8 (Chr12: 4556353–4556502), coding for one of the DNA binding domains in the RAD51AP1 protein (Figure 6C). As RAD51AP1 is involved in both homologous recombination and the response to transcription-replication conflicts^52^^,^^53^^,^^54^^,^^55^ and since the loss of RAD51AP1 has been associated with reduced S-phase checkpoint activation,^56^ we reasoned that RAD51AP1 could be a key factor linking PTBP1 deficiency to altered drug response. To assess how impaired splicing may affect RAD51AP1 levels and function, MCF10A EditR and PTBP1 deficient clone 4.1 cells were either mock-treated or treated with CDC7i or MMC for 24 h before harvesting. Lysates were then fractionated into soluble and chromatin-enriched samples and analyzed by western blot. We found that most of RAD51AP1 was detected in the chromatin-enriched fraction as an immunoreactive band running at ∼33 kDa, however three additional bands with slower migration, possibly because of post-translational modifications,^57^^,^^58^^,^^59^ were also seen in the chromatin and soluble fractions (Figure 6D). The overall levels of RAD51AP1 species were strongly reduced in PTBP1-deficient cells and their distribution was not affected by drug treatment, consistent with the hypothesis that exon skipping leads to the synthesis of a truncated and likely unstable protein (Figure 6D).

**Figure 7:**
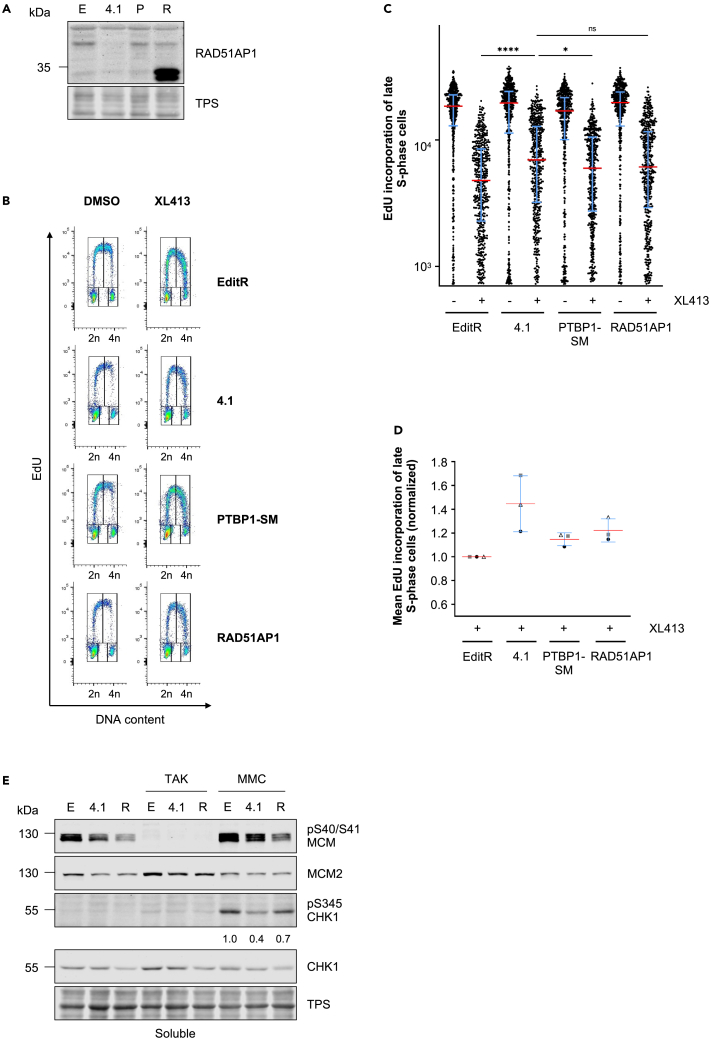
Overexpression of RAD51AP1 partially restores replication control and checkpoint signaling in PTBP1-deficient cells (A) MCF10A EditR (E), PTBP1 mutant clone 4.1 (4.1), PTBP1-SM (P) and RAD51AP1 (R) cells were harvested and soluble protein fractions were prepared. Samples were analyzed by immunoblotting for RAD51AP1 and total protein staining (TPS) was used as loading control. Blot is representative of three independent experiments. (B) MCF10A EditR, PTBP1 mutant clone 4.1, PTBP1-SM and RAD51AP1 cells were treated with DMSO or 20 μM XL413 for 24 h. For flow cytometry analysis, cells were labeled with 10 μM EdU for 30 min before harvesting. Representative images from one of three independent experiments are shown. (C) Fluorescence intensity, proportional to EdU incorporation, in late S-phase cells for representative experiment described in B. Red lines indicate the medians and blue lines show the interquartile range extending from the 25^th^ to the 75^th^ percentile for 510 cells. Statistical analysis was performed using one-way ANOVA, with Tukey’s multiple comparison test (ns, not significant; ∗p < 0.05; ∗∗∗∗p < 0.0001). (D) Mean fluorescence intensity in late S-phase cells of three independent experiments expressed as a ratio relative to 20 μM XL413-treated samples. Red and blue lines show the means ± SDs. (E) MCF10A EditR (E), clone 4.1 (4.1), and RAD51AP1 (R) were treated with 0.625 μM TAK-931 or 200 nM MMC for 24 h. Soluble fractions were prepared and analyzed by immunoblotting. TPS was used as loading control. Numbers represent quantification of pS345 CHK1 signal normalized to TPS and relative to EditR cells treated with MMC. Blots are representative of three independent experiments.

To determine whether the loss of checkpoint signaling and DNA synthesis restraint in PTBP1-deficient cells can be attributed to defective RAD51AP1 function, we ectopically expressed RAD51AP1 in MCF10A PTBP1 mutant clone 4.1 cells (RAD51AP1 cells). These cells expressed high levels of unmodified RAD51AP1 compared to EditR cells and MCF10A PTBP1 mutant clone 4.1 reconstituted with a functional PTBP1 allele (PTBP1-SM), whereas modified forms of RAD51AP1 showed similar levels to both MCF10A EditR and PTBP1-SM cell lines (Figure 7A). Using flow cytometry-based analysis, we determined the importance of RAD51AP1 in restraining DNA synthesis following CDC7i treatment. Similar to PTBP1-SM cells, RAD51AP1 cells displayed reduced EdU incorporation in late S-phase cells compared to MCF10A PTBP1 mutant clone 4.1, suggesting at least a partial re-sensitization of both cell lines to CDC7 inhibition (Figures 7B–7D). Similarly, RAD51AP1 expression in PTBP1-deficient cells partially rescues ATR activation, as demonstrated by the increased phosphorylation of CHK1 following MMC treatment (Figure 7E).

Thus, we can conclude that PTBP1 modulates the response to CDC7 inhibition and replication stress in part through splicing of RAD51AP1, enforcing DNA synthesis inhibition and stimulating ATR signaling.

## Discussion

In this work, we define the relevance of the ATR driven checkpoint response to determine the antiproliferative activity of CDC7is. Careful titration of CDC7 and ATR inhibitors reveals that when substantial levels of inhibition of both kinases is reached, cells are unlikely to complete DNA synthesis within S-phase and, in the absence of checkpoint control, undergo premature and lethal mitosis, which was previously reported by us and others.^32^^,^^33^

However, we find two conditions in which ATRis and CDC7is show antagonism: the first condition is when low levels of CDC7i suppress the antiproliferative effects of high doses of ATRi. Fork instability and collapse is a feature in cells with impaired ATR signaling^60^^,^^61^ and is substantially driven by nucleases acting on unprotected ssDNA.^62^^,^^63^ We have shown that CDC7 promotes MRE11 activity at forks and that CDC7 inhibition suppresses fork collapse induced by long treatment with hydroxyurea. Intriguingly, in the same study we have seen that CDC7 inhibition reduces H2AX phosphorylation in ATRi-treated cells in absence of other replication interfering agents,^32^ suggesting CDC7 inhibition may also partially suppress fork collapse occurring in ATRi-treated cells, thus reducing DNA damage and loss of viability upon ATR inhibition (ref. 32 and Figure 1).

The second condition is when low doses of ATRi, which per se have no visible effects on the cell cycle, counteract the antiproliferative effects of high levels of CDC7i. This effect is mediated by the unleashing of origin firing even in the presence of CDC7is, thus accelerating the progression of cells through S-phase. PTBP1 loss of function, similarly to ETAA1 depletion, mimics this second condition allowing additional origin firing and cells to replicate more efficiently in presence of CDC7is.

Phenotypically, PTBP1 loss of function impairs RPA recruitment on chromatin and RPA focal formation, which explains why ATR signaling, which is dependent on RPA-coated ssDNA,^20^ is deficient. Impaired RPA recruitment could be caused by several reasons, either by fewer forks being generated, which is unlikely as ATR impairment promotes origin firing and fork generation, by reduced fork stalling or by defective processing of forks. We suggest that a combination of the last two may occur as DNA fiber analyses show normal levels of origin firing and fork progression in parental and PTBP1-deficient cells. Consistent with problems in DNA replication, PTBP1-deficient cells show increased 53BP1 G1 nuclear bodies and micronuclei, which are a hallmark of genome instability,^48^^,^^64^ although we cannot exclude that aberrant mitotic events also contribute to the generation of micronuclei in these cells.

Our analysis identifies RAD51AP1 as a target of PTBP1 regulation of pre-mRNA splicing and indeed ectopic expression of RAD51AP1 partially rescues checkpoint deficiency of PTBP1 defective cells. *In vitro* RAD51AP1 facilitates RAD51 D-loop formation, thus it is possible that RAD51AP1 deficiency alters the dynamics/residence time of RPA and RAD51 on ssDNA at stalled forks.^52^^,^^53^^,^^65^ Recent work has shown that at telomeres RAD51AP1 also stabilizes R-loops, thus promoting the formation of G4 DNA structures, which can then promote break-induced replication and alternative lengthening of telomeres in the absence of telomerase.^55^^,^^66^ By analogy, formation and stabilization of R-loops by RAD51AP1 may also occur in other regions of the genome, thus providing obstacles to the replication machinery and increasing the dependency on ATR signaling. Such a hypothesis is consistent with previous work in DT-40 chicken cells showing that in unchallenged S-phase RAD51AP1 knock-out alters progression of replication forks and increases origin usage, which also in this system could be because of defective ATR signaling.^56^ Furthermore, proteomics studies using proximity-based biotinylation have identified RIF1 and TOPBP1, which are important for determining the efficiency of origin firing and ATR activation, as part of the RAD51AP1 proximal interactome.^66^ This may suggest an alternative mechanism to couple the events occurring at replication forks with late/dormant origin activation. As the reintroduction of RAD51AP1 only partially suppresses the replication and checkpoint phenotypes of PTBP1 deficiency, other mechanisms are involved and we speculate that, on fork stalling, further defects in signal generation and amplification could be because of misfunction of nucleases, such as MRE11, and the imbalance in the ratio of the ATR kinase activating subunits (Figure S7).

Although this work positions PTBP1 as a novel and important factor in the replication stress response, so that the efficacy of CDC7is in suppressing DNA synthesis and proliferation is profoundly diminished in PTBP1-deficient cells within the first cell cycle, it did not escape our attention that PTBP1 also has an important role in driving cellular senescence, which can result from either excessive or prolonged DNA damage or replication stress. CDC7 inhibition can drive senescence in several cell types^67^^,^^68^ and CDC7 inhibition in combination with agents that eliminate senescent cells was shown to have a very profound anti-tumor activity specifically in p53 mutated liver cancer models, suggesting a possible strategy to target these very aggressive tumors for which there is still a major unmet medical need.^67^ As a note of caution, if such a therapeutic strategy is pursued, based on this study, it is predicted that resistance and relapse may arise by pre-existing and acquired mutations in PTBP1 or in factors controlling PTBP1 expression.

### Limitations of this study

Though our mechanistic study positions PTBP1 as an important factor in the replication stress response, this study has some limitations. Our data were solely generated in MCF10A cells where PTBP1 deficiency had a broad effect on mRNA splicing; in other cell lines PTBP1 deficiency may differently affect gene expression, thus the phenotypes described here for PTBP1 deficiency could be either more or less penetrant. In addition, this study does not include preclinical experimentation to conclude that the anti-tumor activity of CDC7 inhibitors is decreased in an *in vivo* setting. Here, we limited our investigation to RAD51AP1 as downstream effector of PTBP1’s replication and checkpoint phenotype. However, additional factors contributing to these phenotypes are likely to exist, and their identification would complete the data presented in this study.

## STAR★Methods

### Key resources table

**Table:** 

REAGENT or RESOURCE	SOURCE	IDENTIFIER
**Antibodies**		

Anti-53BP1 rabbit polyclonal	Novus Biologicals	Cat# NB100-304; RRID: AB_10003037
Anti-ATR pT1989 rabbit polyclonal	GeneTex	Cat# GTX128145; RRID:AB_2687562
Anti-beta-Tubulin mouse monoclonal	Millipore	Cat# 05-661; RRID: AB_309885
Anti-BrdU (BU1/75 (ICR1)) rat monoclonal	ThermoFisher	Cat# MA1-82088; RRID: AB_927214
Anti-BrdU (B44) IgG1 mouse monoclonal	BD biosciences	Cat# 347580; RRID: AB_10015219
Anti-CHK1 pS345 (133D3) rabbit monoclonal	Cell Signaling Technology	Cat# 2348; RRID:AB_331212
Anti-CHK1 (G4) mouse monoclonal	Santa Cruz Biotechnology	Cat# SC-8408; RRID:AB_627257
Anti-MCM2 rabbit polyclonal	in house previously described (Rainey et al. 2017)^26^	N/A
Anti-MCM2 pS40/41 rabbit polyclonal	in house previously described (Rainey et al. 2017)^26^	N/A
Anti-hnRNP I (Anti-PTBP1) (SH54) mouse monoclonal	Santa Cruz Biotechnology	Cat# SC-56701; RRID:AB_783822
Anti-hnRNP I (Anti-PTBP1) (A-4) mouse monoclonal	Santa Cruz Biotechnology	Cat# SC-515282
Anti-RPA2 (Ab-3) mouse monoclonal	Calbiochem	Cat# NA19L; RRID:AB_565123
Anti-RAD51AP1 rabbit polyclonal	Sigma-Aldrich/Merck	Cat# HPA051499; RRID:AB_2681508
Anti-RAD51 rabbit polyclonal	Santa Cruz Biotechnology	Cat# SC-8349; RRID:AB_2253533
IRDye 800CW goat anti-rabbit IgG antibody	LI-COR Biosciences	Cat# 926-32211; RRID:AB_621843
IRDye 800CW goat anti-mouse IgG antibody	LI-COR Biosciences	Cat# 926-32210; RRID:AB_621842
Chicken anti-rat Alexa Fluor 488	ThermoFisher	Cat# A21470; RRID: AB_2535873
Goat anti-rabbit IgG Alexa Fluor 546	ThermoFisher	Cat# A11010; RRID AB_2534077
Goat anti-mouse IgG Alexa Fluor 546	ThermoFisher	Cat# A11003; RRID: AB_2534071
Goat anti-mouse IgG1 Alexa Fluor 546	ThermoFisher	Cat# A21123; RRID:AB_2535765

**Bacterial and virus strains**		

NEB® Stable Competent *E. coli*	New England BioLabs	Cat# C3040

**Chemicals, peptides, and recombinant proteins**		

4′,6-Diamidino-2-phenylindole dihydrochloride (DAPI)	Sigma-Aldrich/Merck	Cat# D9542
5-Ethynyl-20-deoxyuridine (EdU)	Berry & associates	Cat# PY 7562
6-Carboxyfluorescein-TEG azide	Berry & associates	Cat# FF 6110
AF647-TEG-azide	Jena Biosciences	Cat# CLK 1299-1
AZD6738	MedChemExpress	Cat# HY-19323
Benzonase (≥250 units/μl)	Sigma-Aldrich/Merck	Cat# E1014
Camptothecin	Sigma-Aldrich/Merck	Cat# C9911
Cholera toxin	Sigma-Aldrich/Merck	Cat# C8052
Cis-platin	Sigma-Aldrich/Merck	Cat# P4394
Crystal violet	Sigma-Aldrich/Merck	Cat# C0775
Human EGF	Peprotech	Cat# AF-100-15
Etoposide	Sigma-Aldrich/Merck	Cat# E1383
Fast Green FCF	Sigma-Aldrich/Merck	Cat# F7252
Fisher Bioreagents Phosphatase Inhibitor Cocktail I	ThermoFisher	Cat# 12821650
Fisher Bioreagents Protease Inhibitor Cocktail III	ThermoFisher	Cat# 12831640
G418 disulfate salt	Sigma-Aldrich/Merck	Cat# A1720
Horse serum	Sigma-Aldrich/Merck	Cat# H1270
Human insulin solution	Sigma-Aldrich/Merck	Cat# I9278
JetPrime	Polyplus transfection	Cat# 114-15
Mitomycin C	Sigma-Aldrich/Merck	Cat# M4287
Polybrene (Hexadimethrine bromide)	Sigma-Aldrich/Merck	Cat# H9268
Polyethyleneimine ‘‘MAX’’ (MW 40,000)	Polysciences Inc.	Cat# 24765
Resazurin Sodium Salt	Sigma-Aldrich/Merck	Cat# R7017
RNase A solution	Qiagen	Cat# 1007885
SlowFade Gold Antifade Mountant	ThermoFisher	Cat# S36937
TAK-931	Chemietek	Cat# CT-TAK931
XL413	In house previously described (Rainey et al., 2013)^30^	N/A

**Critical commercial assays**		

RNeasy mini kit	Qiagen	Cat# 74106
SuperScript™ II Reverse Transcriptase kit	Invitrogen	Cat# 18064014
QuikChange II XL Site-Directed Mutagenesis kit	Agilent	Cat# 200521
MN NucleoSpin® Gel and PCR clean-up kit	Macherey-Nagel	Cat# 740609.50

**Deposited data**		

RNA-seq data	This paper	ArrayExpress: E-MTAB-12578
Source data for main and supplemental figures	This paper	Mendeley Data: https://doi.org/10.17632/7rgn4p4cvp.1

**Experimental models: Cell lines**		

Human: MCF10A	ATCC	Cat# CRL-10317; RRID:CVCL_0598
Human: MCF10A EditR (Constitutive Cas9 expression)	Previously described (Rainey et al., 2017)^26^	N/A
Human: MCF10A PTBP1 4.1	This paper	N/A
Human: MCF10A PTBP1 6.6	This paper	N/A
Human: MCF10A PTBP1-SM	This paper	N/A
Human: MCF10A RAD51AP1	This paper	N/A
Human: Lenti-X™ 293T	TaKaRa	Cat# 632180

**Oligonucleotides**		

siRNA, primer and sgRNA oligonucleotide sequences	Table S4	N/A

**Recombinant DNA**		

LRG (Lenti_sgRNA_EFS_GFP)	Addgene; previously described (Shi et al., 2015)^69^	RRID:Addgene_65656
PTBP1-g4-LRG (Constitutive expression of PTBP1-guide RNA4 and GFP)	This paper	N/A
PTBP1-g6-LRG (Constitutive expression of PTBP1-guide RNA6 and GFP)	This paper	N/A
Ready-to-Use Lentiviral Packaging Plasmids Mix	Cellecta	Cat# CPCP-K2A
pCDH-EF1-MCS-IRES-Neomycin Cloning and Expression Lentivector	System Biosciences	Cat# CD533A-2
pCDH-EF1-PTBP1-SM-IRES-Neomycin	This paper	N/A
pCDH-EF1-RAD51AP1-IRES-Neomycin	This paper	N/A

**Software and algorithms**		

ImageJ	ImageJ (Schindelin et al., 2012)^70^	https://imagej.nih.gov/ij/
FlowJo 10.0.7	FlowJO	https://www.flowjo.com
Image Studio 2.0.38	LI-COR Biosciences	https://www.licor.com
Image Studio Lite 5.2.5	LI-COR Biosciences	https://www.licor.com
GraphPad Prism 9.5.0 for windows	GraphPad	https://www.graphpad.com
Empiria 1.3.0.83	LI-COR Biosciences	https://www.licor.com
Harmony® high-content analysis software	PerkinElmer	Cat# HH17000001
TIDE version 3.3.0	Previously described (Brinkman et al., 2014)^71^	https://tide.nki.nl
SynergyFinder 3.0	Previously described (Ianevski et al., 2022)^72^	https://synergyfinder.fimm.fi
Integrative Genomics Viewer (IGV) software	Previously described (Thorvaldsdóttir et al., 2013)^73^	https://igv.org/app/
DexSeq (Bioconductor version)	Bioconductor; Previously described (Anders et al., 2012)^74^	https://bioconductor.org/packages/release/bioc/html/DEXSeq.html

**Other**		

Nitrocellulose	GE Healthcare	Cat# 10600001
BD AccuriC6 flow cytometer	BD Biosciences	Custom
BD FACS Canto II flow cytometer	BD Biosciences	Custom
Odyssey Infrared Imaging System	LI-COR	Custom
Operetta CLS High Content Analysis System	PerkinElmer	Custom

### Resource availability

#### Lead contact

Further information and requests for resources and reagents should be directed to and will be fulfilled by the lead contact, Corrado Santocanale (corrado.santocanale@universityofgalway.ie).

#### Materials availability

All reagents generated in this study will be made available on request, but we may require a payment and/or a completed Materials Transfer Agreement, if there is potential for commercial application.

### Experimental model and subject details

#### Cell culture

Cell culture was performed in a Class II Bio-safety cabinet and all cell lines were maintained at 37°C in a humidified atmosphere containing 5% CO_2_. Cell counts and viability were determined using a countess (Invitrogen) or LUNA II cell counter (Logos biosystems) and trypan blue exclusion. If not specified otherwise, all culture media and reagents were obtained from Merck. MCF10A cell line (human, female origin) was purchased from ATCC and authenticated via genome sequencing. MCF10A EditR cells (stably expressing Cas9) were generated as previously described in Rainey et al., 2017.^26^ MCF10A PTBP1 mutant clone 4.1 and 6.6 were derived via CRISPR/Cas9 genome editing from MCF10A EditR cells and monoclonal expansion following a competitive proliferation assay described in method details. Mutation of PTBP1 was verified via targeted PCR and Sanger sequencing. MCF10A PTBP1-SM and MCF10A RAD51AP1 cells were generated for this publication as detailed in method details from MCF10A PTBP1 mutant clone 4.1. MCF10A cells and derivatives were cultured using DMEM supplemented with 5% (v/v) horse serum, 25 ng/ml cholera toxin, 10 μg/ml insulin, 20 ng/ml epidermal growth factor (Peprotech), 500 ng/ml hydrocortisone, 50 U/ml penicillin and 50 μg/ml streptomycin. MCF10A PTBP1-SM and MCF10A RAD51AP1 cells were additionally kept in 0.5 mg/ml G418 for selection. Lenti-X™ 293T cell line (human, female origin) were purchased from TaKaRa and used without further authentication. Routine culture was performed using DMEM + GlutMAX™-I (ThermoFisher) supplemented with 10% (v/v) fetal bovine serum, 50 U/ml penicillin and 50 mg/ml streptomycin.

### Method details

#### Drugs treatments

If not otherwise indicated, XL413 (synthesized in house) was used at a concentration of 20 μM and TAK-931 (Chemietek) was used at a concentration of 0.625 μM. Etoposide was used at 10 μM, camptothecin at 50 nM, *cis*-platin at 5 μM, and MMC at 200 nM (all acquired from Merck).

#### Competitive proliferation assay

To assess the effects of gene editing *PTBP1* with PTBP1-sgRNA4 and PTBP1-sgRNA6, we adapted a competitive proliferation assay previously described in Rainey et al., 2020.^19^ Oligonucleotides coding for sgRNAs were cloned into BsmBI restriction site of the LRG vector (Lenti_sgRNA_EFS_GFP; Addgene)^69^ to generate PTBP1-g4-LRG and PTBP1-g6-LRG plasmids. MCF10A EditR cells were plated at 120,000 cells per well in 6-well plates. After 24 hours cells were transduced with viral particles containing either empty LRG, PTBP1-g4-LRG or PTBP1-g6-LRG at a Multiplicity of Infection (MoI) of 0.1. 24 hours after transduction, the culture media was changed and after an additional 24 hours cells were harvested and divided into two samples. The first sample was fixed in 4% (w/v) PFA in PBS for 20 min at room temperature before being washed once and resuspended in PBS. Cells were analysed with an AccuriC6 flow cytometer to determine cell count and levels of GFP expression as a readout for transduction efficiency. Cells were replated at 80,000 cells/well in the presence of 20 μM XL413. Samples were incubated over 16 days in the continued presence of 20 μM XL413 with a change of media every 4 days. Alternatively, to assess the ability of PTBP1 mutant clone 4.1 to resist long-term CDC7i treatment, MCF10A EditR and GFP expressing PTBP1 mutant clone 4.1 or RAD51AP1 expressing cells were harvested, counted and a sample was fixed as described above. The GFP expression was analysed using a BD FACS Canto II flow cytometer. Based on cell counts, 40,000 cells of MCF10A EditR and PTBP1 mutant clone 4.1 cells were combined and plated per well in the presence of XL413 or TAK-931 to achieve approximately 50% GFP-positive cells per well. Plates were incubated in a humid chamber for 8 days in the continued presence of XL413 or TAK-931. Analysis of GFP expression and replating was conducted as described once cells reached ∼80% confluency.

#### Establishing monoclonal cell lines with PTBP1 mutations

MCF10A EditR cells expressing PTBP1-sgRNA4 and PTBP1-sgRNA6 from the competitive proliferation assay were subject to limited dilution and incubated in 200 μl of complete media in 96-well plates for 10-14 days. Cells from wells that contained only single colonies were expanded and genotyped to assess PTBP1 mutational status. For screening, adherent cells in 96-well plates were washed with PBS and lysed in 50 μl of lysis buffer (10 mM Tris-HCl pH 7.5, 10 mM EDTA, 10 mM NaCl, 0.5% (w/v) N-Lauryl sarcosine, 10 μg/ml proteinase K and 20 μg/ml glycogen) for 2 hours at 60°C. Genomic DNA was precipitated by adding 3x volumes of 150 mM NaCl in 96% (v/v) EtOH before mixing and incubation at room temperature for 30 min. DNA was pelleted by centrifugation (15 min, 800 x g), washed with 70% (v/v) EtOH and re-pelleted prior to air drying and resuspension in 100 μl TE buffer (10 mM Tris-HCl pH 7.5, 1 mM EDTA).

5 μl genomic DNA were used in PCR reactions using Taq DNA Polymerase and screening primers (Table S4: PTBP1-sg4 fwd/rev, PTBP1-sg6 fwd/rev). PCR products were purified with MACHEREY-NAGEL NucleoSpin® Gel and PCR clean-up kit and sequenced using PTBP1-sg4 fwd or PTBP1-sg6 fwd primer (Table S4) by Eurofins genomics. The web-based tool TIDE (https://tide.nki.nl)^71^ was used to assess genome editing of the *PTBP1* locus. The chromatogram files from a sanger sequencing analysis of a control sample and a test sample were uploaded to TIDE alongside the 20-nucleotide sequence of the small guide RNA used to direct Cas9 to the target genomic locus. Using the quantitative sanger sequence trace data, TIDE analysis discriminated between wild-type, homozygous, heterozygous, and complex genotypes and provided predictions for potential indels at the CRISPR cut site. TIDE analysis indel predictions were confirmed by manual analysis of the Sanger sequencing reads.

#### Generation of lentiviral transfer plasmids

Total RNA was isolated from 1x10^6^ MCF10A cells using the RNeasy mini kit (Qiagen) according to the manufacturer’s instructions. Subsequently, 1 μg of purified total RNA was subject to first-stand cDNA synthesis using Oligo(dT) primers and the SuperScript™ II Reverse Transcriptase kit (Invitrogen). The resulting cDNA library was used as a template for PCR amplification using gene specific primers with adaptors. The primers were designed to amplify PTBP1 (PTBP1-fwd/rev) and RAD51AP1 (RAD51AP1-fwd/rev) from start to stop codon and add a NotI restriction site for cloning (Table S4). PCR products were cloned into the Not1 restriction sites of the lentiviral transfer plasmid pCDH-EF1-MCS-IRES-Neomycin (System Biosciences) and inserts were verified by Sanger sequencing (Eurofins genomics). To generate a sgRNA4-resistant variant of the cloned PTBP1 coding sequence, primers for site-directed mutagenesis (PTBP1-SDM fwd/rev) were designed (Table S4). These primers generate a silent mutation in the PAM site adjacent to the target sequence for sgRNA4 (3’-CTCCGTGTTCATCTCGATGA-5’). Site-directed mutagenesis was performed using the QuikChange II XL kit (Agilent) according to the manufacturer’s instructions. The expected sgRNA4-resistant PTBP1-variant with the silent mutation (PTBP1-SM) was verified by Sanger sequencing. The lentiviral transfer plasmids: pCDH-EF1-PTBP1-SM-IRES-Neomycin and pCDH-EF1-RAD51AP1-IRES-Neomycin were used for small-scale lentiviral packaging.

#### Small scale lentiviral packaging

Lenti-X™ 293T cells were seeded at 140,000 cells per well in a 6-well plate. After 24 hours, transfections were performed using a 1:2 ratio (w/v) of DNA to polyethyleneimine “MAX” MW 40,000 (1 mg/ml, Polysciences). 2 μg of Lentiviral Packaging Plasmids (Cellecta) and 0.4 μg lentiviral transfer plasmids were added to 100 μl of 150 mM NaCl prior to mixing with polyethyleneimine (4.8 μl) that was diluted separately in 100 μl of 150 mM NaCl. Reagents were vortexed and incubated at room temperature for 20 min before dropwise addition to cells. Media was changed after 24 hours and transfected Lenti-X™ 293T cells were allowed to produce virus for a further 24 hours. 2 ml of media was harvested, centrifuged (1,500 rpm for 5 min) and passed through a 0.45 μm sterile filter disc before being used for transduction of target cell lines.

#### Transduction of transgene expression vectors

MCF10A PTBP1-deficient clone 4.1 cells were seeded at 120,000 cells per well in a 6-well plate and after 24 hours were transduced with viral supernatant in the presence of polybrene (5 μg/ml). After 24 hours, cells that had undergone transduction were selected by adding fresh media containing G418 (0.5 mg/ml). Expression of the transgenes in these polyclonal cell populations was confirmed by western blotting.

#### siRNA transfections

MCF10A cells were seeded at 80,000 cells per well in a 6-well plate and transfected 24 hours after plating. siRNA transfections were performed using JetPRIME transfection reagent (Polyplus Transfection). For each individual well, siRNAs (50 nM final concentration) were prepared by mixing with JetPrime buffer (200 μl) and JetPrime reagent (4 μl) and the mixture was incubated for 15 min at room temperature prior to dropwise addition to the cells. Following a 5 hours incubation at 37°C, 5% CO_2_ the culture media was exchanged for fresh, incubator-equilibrated media and cells were returned to the incubator. In this study, treatment of siRNA transfected cells was performed 48 hours after transfection.

Protein depletion was confirmed by immunoblotting analysis. Oligonucleotide sequence information for custom synthesized siRNAs (obtained from Merck) is summarized in Table S4.

#### Proliferation assay with crystal violet

MCF10A cells were seeded at 50,000 cells per well in a 6-well plate and treated 24 hours after plating. Cells were allowed to proliferate for 72 hours in media containing DMSO, 10 μM XL413 or 20 μM XL413. Media was discarded and plates were washed with PBS and allowed to dry completely. Cells were fixed by the addition of 2 ml 100% methanol and incubation for 30 min at room temperature. The methanol was discarded and the plates were air dried for 10 min. Cells were stained with 3 ml crystal violet solution (0.5% (w/v) crystal violet in 25% (v/v) methanol) for 30 min at room temperature and washed with H_2_O until any excess staining solution was removed. Plates were left to dry overnight. The plates were analysed using the Odyssey Infrared Imaging System and the intensity of cell stain was calculated using Image Studio software (LI-COR Biosciences). All values were normalised to corresponding DMSO controls.

#### 96-well plates resazurin reduction assays

MCF10A cells were seeded at 2,500 cells per well in 96-well plates. Each well already contained 50 μl of inhibitors either alone or in combination. Each experiment was performed in technical triplicates. The effect of the CDC7 inhibitor TAK-931 was tested at six different concentrations using a 2-fold dilution that ranged from 5 to 0.0195 μM. The effect of the CDC7 inhibitor XL413 was tested at different concentrations using a 2-fold dilution that ranged from 40 to 2.5 μM or alternatively from 80 to 0.3125 μM. The effect of the ATR inhibitor, AZD6738, was tested at different concentrations using a 2-fold dilution series that ranged from 10 to 0.0391 μM. After 72 hours of drug treatment, the resazurin reduction assays were performed by addition of 0.56 mM resazurin sodium salt in PBS to each well to a final concentration of 93 μM. Plates were incubated for 6 hours at 37°C with 5% CO_2_ and fluorescence intensity was determined using a Victor 3V 1420 multilabel counter plate reader with 530 nm excitation and 595 nm emission. Background from wells that contained only media and resazurin solution was subtracted and fluorescence intensity values were normalised to values from untreated controls to determine the relative surviving fraction. The half-maximal inhibitor concentration values (IC_50_) were determined using GraphPad 9.5.0 by plotting the relative survival fraction of three biological repeats against the Log10 inhibitor concentration and using a nonlinear regression (curve fit) and log(inhibitor) versus normalized response-variable slope equation.

Additionally, the average surviving fraction for each individual and combination dose across three biological repeats was analysed using SynergyFinder 3.0 for interactive analysis and visualization of multi-drug and multi-dose combination response data (https://synergyfinder.fimm.fi).^72^ Data was submitted to SynergyFinder as % viability and the recommended default options were used for the four-parameter logistic regression (LL4) curve fitting algorithm to fit single-drug dose-response curves. To investigate the degree of combination synergy or antagonism, the Zero interaction potency (ZIP) model was used to compare the change in the potency of the dose-response curves between individual drugs and their combinations. 2D and 3D synergy maps were downloaded from the SynergyFinder website and highlight synergistic and antagonistic dose regions in red and green colours, respectively. Delta scores obtained from the interactive 3D synergy plot were used as a guide to describe the interaction between the drug combinations: Antagonistic (δ score <−10), additive (δ score from −10 to 10) or synergistic (δ score >10).

#### Flow cytometry

To analyse cell cycle distribution and rate of DNA synthesis, MCF10A cells were seeded at 160,000 per well in 6-well plates. Following treatment with indicated inhibitors or DMSO, nascent DNA was labelled by incubating cells with 10 μM EdU for 30 min at 37°C prior to harvesting. Cells were washed with PBS, which if not specified otherwise involves centrifugation at 400 x g for 5 min at 4°C and the removal of the supernatant from the cell pellet. Samples were fixed by resuspension in 0.3 ml PBS and dropwise addition of 0.7 ml 100% ethanol while vortexing followed by incubation for at least 1 hr at -20°C. After a PBS wash, cells were washed with 1% (w/v) BSA/PBS and incorporated EdU was labelled by incubating cells in click reaction buffer (PBS containing 10 mM Sodium-L-ascorbate, 2 mM Copper-II-Sulfate and 10 μM AF647-TEG-azide (Jena, CLK 1299-1) or alternatively 10 μM 6-Carboxyfluorescine-TEG-azide) for 30 min at room temperature protected from light. Cells were then incubated in PBS containing 1% (w/v) BSA and 0.5% (v/v) Tween-20 for 5 min at room temperature followed by an additional wash with PBS. DNA was stained with 1 μg/ml DAPI in 1% (w/v) BSA/PBS and to reduce RNA interference 0.5 μg/ml RNase A was added to each sample until measurement. Fluorescence intensity data for DAPI (405_450_50 nm) and AF647-TEG-azide (633_660_20 nm) or alternatively 6-Carboxyfluorescine (488_530_30 nm) were acquired for 10,000 single cells on a BD FACS Canto II and analysed using FlowJo 10.0.7 software. To perform analysis of fluorescence intensity, proportional to EdU incorporation, for individual cells, gates were applied on DAPI-EdU biparametric dot plots to select for EdU-positive late S-phase cells using FlowJo. Fluorescence intensity (488_530_30-H or 633_660_20-H) values per cell were exported and plotted as scatter dot plots using GraphPad prism 9.5.0. For the comparison of fluorescence intensity across three biological repeats, mean fluorescence intensity of each sample was normalised to the mean of XL413-treated MCF10A EditR cells within the respective experiment. Data were plotted as a scatter dot plot using GraphPad prism 9.5.0.

#### Protein samples preparation

For chromatin fractionation MCF10A cells were seeded at 160,000 per well in 6-well plates and following treatment with indicated inhibitors or DMSO, cells were harvested, washed once with PBS and lysed in CSK buffer (10 mM PIPES pH 6.8; 300 mM sucrose; 100 mM NaCl; 1.5 mM MgCl_2_; 0.5% (v/v) Triton X-100; 1 mM ATP; 1 mM DTT; 1 mM sodium orthovanadate; 2 mM N-ethylmaleimide; Phosphatase Inhibitor Cocktail I and Protease Inhibitor Cocktail III (ThermoFisher)) for 10 min on ice. Samples were centrifuged at 1,000 x g for 4 min at 4°C, and the supernatant was transferred to a new reaction tube (soluble fraction). The pellet was washed using CSK buffer (2x volume of soluble fraction), centrifuged at 1,000 x g for 4 min at 4°C and the supernatant was discarded. Pellets were resuspended in CSK buffer containing benzonase (125 U/ml) and incubated for 30 min on ice. Samples were denatured for 5 min at 95°C in 1x Laemmli buffer (Chromatin-enriched fraction). Protein concentration of the soluble fraction was determined by Bradford assay, and required amount of protein was denatured for 5 min at 95°C in 1x Laemmli buffer.

For whole cell extracts cells were harvested, washed with PBS, and resuspended in RIPA buffer (50 mM Tris-HCl pH 7.5; 150 mM NaCl; 1 mM MgCl_2_; 0.1% (w/v) sodium dodecyl sulfate (SDS); 0.5% (w/v) sodium deoxycholate; 1 mM EDTA; 1 mM sodium orthovanadate; 2 mM n-ethylmaleimide; Phosphatase Inhibitor Cocktail I and Protease Inhibitor Cocktail III) containing benzonase (125 U/ml) and incubated for 30 min on ice. Protein content was determined using bicinchoninic acid (BCA) assay, and 20 μg of extract was denatured for 5 min at 95°C in 1x Laemmli buffer. Proteins were separated by SDS-PAGE.

#### Immunoblotting

Proteins were transferred onto 0.2 μm pore size nitrocellulose membranes using a wet blot transfer system (Biorad). Proteins on membranes were stained with fast green (0.0001% (w/v) fast green in 0.1% (v/v) acetic acid) for 5 min as a total protein stain (TPS) and were analysed on the Odyssey infrared imaging system at 680 nm (LI-COR Biosciences). Membranes were de-stained with 0.1 M NaOH in 30% (v/v) methanol for 10 min and washed three times in ddH_2_O for 5 min at room temperature. Membranes were blocked in 3% (w/v) skim milk (Sigma) in TBS-T (20 mM Tris-HCl pH 7.5, 150 mM NaCl, 0.05% (v/v) Tween-20) for 1 hr at room temperature. Membranes were incubated in primary antibody diluted in blocking buffer overnight at 4°C followed by three washes in TBS-T for 10 min each. Secondary antibodies were diluted in blocking buffer and membranes were incubated for 1 hr at room temperature (protected from light) followed by three washes in TBS-T for 10 min at room temperature. Signals were acquired using the Odyssey infrared imaging system and analysed using Image Studio 2.0.38 and Empiria software 1.3.0.83 (LI-COR).

Primary antibodies were diluted in 3% skim milk/TBS-T: CHK1 (sc8408; Santa Cruz Biotech; 1:1000), RPA2 (NA19L, Calbiochem; 1:1000), hnRNP I/PTBP1 (sc56701; Santa Cruz; 1:1000), hnRNP I/PTBP1 (sc515282; Santa Cruz; 1:1000), RAD51AP1 (HPA051499; Merck; 1:1000), RAD51 (sc8349; Santa Cruz; 1:1000) and MCM2 (in house: 1:3000) or 1% BSA/TBS-T: pT1989 ATR (GTX128145; GeneTex; 1:1000), pS345 CHK1 (2348, CST, 1:1000) and pS40/S41 MCM2 (in house; 1:3000). IRDye secondary antibodies (LI-COR): 800CW goat anti-rabbit (926-32211; LI-COR Biosciences; 1:10,000) and 800CW goat anti-mouse (926-32210; LI-COR Biosciences; 1:10,000) were diluted in the same buffer as the primary antibody.

#### Fluorescence microscopy

MCF10A cells were seeded at a density of 150,000 cells per well in a 6-well plate on poly-L-lysine coated coverslip. Following drug treatment, coverslips were washed once with PBS and cells were fixed in PBS containing 4% (w/v) PFA for 10 min at room temperature. For the detection of nuclear RPA2, a cytoplasmic extraction with CSK buffer (see section: protein samples preparation) was performed for 15 min on ice prior to PFA fixation. Following the fixation step, samples were washed three times with PBS to remove residual PFA. Cells were permeabilized with PBS-TX (0.1% (v/v) Triton X-100 in PBS) for 20 min at room temperature or alternatively for 53BP1 detection, cells were permeabilized with PBS-TX (0.125% (v/v) Triton X-100 in PBS) for 3 min at room temperature, followed by incubation for 30 min in blocking buffer (PTBP1, RPA2, beta-Tubulin staining: 10% (v/v) FBS, 0.5% (w/v) BSA in PBS-TX; 53BP1 staining: 1%.(w/v) BSA in PBS) at room temperature. Cells were incubated for 1 hr at 37°C with either mouse anti-PTBP1 (sc56701; Santa Cruz; 1:500), mouse anti-beta-Tubulin (#05-661; Millipore; 1:2000), rabbit anti-53BP1 (NB100-304; Novus Biologicals; 1:200) or mouse anti-RPA2 (NA19L, Calbiochem; 1:500) primary antibody diluted in blocking buffer. Following three washes with PBS-TX or PBS for 53BP1 detection, coverslips were incubated for 1 hr at 37°C with goat anti-mouse AlexaFluor 546 (A11003; ThermoFisher; 1:500) or goat anti-rabbit AlexaFluor 546 (A11010; ThermoFisher; 1:500) secondary antibody diluted in blocking buffer, which additionally contained DAPI (1:600 dilution in PBS of 0.5 mg/ml stock) to stain nuclei. Cover slips were washed three times in PBS-TX or PBS for 53BP1 detection, once in PBS and dipped in ddH_2_O before being mounted onto slides using Slow Fade Gold Antifade Reagent (ThermoFisher).

Microscopy for PTBP1, 53BP1 and beta-Tubulin detection was performed using IX71 Olympus microscope using a 40X objective lens or 60X oil-immersion objective as indicated. Mean fluorescence of nuclear PTBP1 signal was analysed for a minimum of 300 cells per sample in two independent experiments using ImageJ Fiji software.^70^ Background signal was calculated from three independent measurements of empty areas for each picture and subtracted from the PTBP1 fluorescence intensity.

The number of micronuclei per cell were manually counted using DAPI staining. A minimum of 300 cells per sample of three independent experiments were analysed. Due to the uniform staining protocol for PTBP1 and beta-Tubulin, micronuclei counts of both experiments were combined.

53BP1 foci analysis was performed using ImageJ Fiji software. Intensity of the DAPI staining was used to identify cells in G1-phase by their low DNA content compared to S- or G2-phase cells. Low DAPI intensity was defined as less than the mean DAPI intensity of at least 610 cells for each sample. 53BP1 nuclear bodies were counted with ImageJ Fiji using the find maxima function and selection for high intensity foci. 53BP1 foci for 300 G1-phase cells were analysed for each condition and three independent experiments.

Mitotic aberrations were analysed manually by identifying mitotic cells and counting the following aberrant structures in samples with DAPI (DNA) and beta-Tubulin staining (microtubules; mitotic spindle): Multipolar spindles/spindle defects, chromatin bridges (broken and intact), unaligned or lagging chromosomes, chromosome fragments. At least 150 mitotic cells were analysed per sample in four independent experiments.

RPA2 analysis and microscopy were performed using the 60X objective lens on the Operetta CLS High Content Analysis System (PerkinElmer, London, UK). Programmed analysis using Harmony analysis software 3.1.1 was used to quantify the number of nuclear RPA2 foci per cell for a minimum of 200 cells per sample in three independent experiments. The analysis sequence used was as follows: identify nuclei based on DAPI intensity, exclude border cells, exclude doublet cells, detect foci based on signal to background ratio, output foci/nucleus counts.

After quantification and analysis, the brightness of representative images was adjusted to the same extent for all samples in an experiment to aid visualisation in figures.

#### DNA fibre spreading

Cells were seeded at a density of 300,000 cells per well in a 6-well plate and allowed to recover overnight. To assess the effect of ATRi on MCF10A EditR cells in the presence of CDC7i, cells were treated with DMSO or 20 μM XL413 and 20 μM CldU for 30 min before the media was exchanged and cells were washed three times with pre-warmed culture media followed by treatment with DMSO, 0.156 μM or 5 μM AZD6738 and labelled with 200 μM IdU for 30 min in the continued presence or absence of XL413. Additionally, one sample was left untreated and unlabelled for later use. Cells were harvested, resuspended in ice-cold PBS, counted, and diluted to 2.5x10^5^ cells/ml. Labelled cells were diluted 1:4 with unlabelled cells and gently mixed. 2.5 μl of the cell solution was removed and placed onto a coverslip before adding 7.5 μl of spreading buffer (0.5% (w/v) SDS, 200 mM Tris-HCl pH 7.4, 50 mM EDTA) and incubating the coverslip at room temperature for 8 min. The coverslips were tilted by 15° to allow the DNA to slowly run down the slide and air dried before being fixed in methanol/acetic acid (3:1) at 4°C overnight. Coverslips were removed from the fixative, incubated in 2.5 M HCl for 1 hr at room temperature to denature the DNA followed by three washes with PBS for 5 min to neutralise the acid. Cover slips were incubated in filter-sterilized blocking buffer (PBS, 1% (w/v) BSA, 0.1% (v/v) Tween-20) for 30 min at room temperature and taken through a series of 30 min primary and then secondary antibody incubations in blocking buffer at room temperature, with two PBS washes and one wash with blocking buffer in between each antibody incubation. The antibodies were used in the following order and concentrations: BrdU (BU1/75) rat monoclonal antibody (MA1-82088; ThermoFisher; 1:100) and Chicken anti-rat Alexa Fluor 488 (A21470, ThermoFisher; 1:300) to detect incorporated CldU. Anti-BrdU (B44) IgG1 mouse monoclonal (347580; BD biosciences; 1:100) and Goat anti-mouse IgG1 Alexa Fluor 546 (A21123, ThermoFisher; 1:300) to detect incorporated IdU. After the final antibody, coverslips were washed three times in PBS and once in ddH_2_O. Coverslips were mounted onto slides using SlowFade Gold Antifade Reagent (ThermoFisher). Images were captured with an IX71-Olympus microscope and 60X oil-immersion objective and analysis was performed manually in ImageJ Fiji software.

#### RNA sequencing

RNA was isolated from MCF10A EditR and PTBP1 mutant clone 4.1 cells using the RNeasy Plus Micro kit (Qiagen). The quality and concentration of the isolated RNA were analysed on a 2100 Bioanalyzer using the Agilent RNA 6000 Nano assay. RNA library preparation, sequencing and bioinformatic analysis was performed by Novogene, UK. A sequencing depth of 70 million paired end reads was used, in non-stranded protocol. The sequencing data was then aligned to the GRCh38 p.13. human reference genome assembly. The normalised expression level of each gene (FPKM value) was calculated. Integrative Genomics Viewer (IGV) software was used to visualise and analyse the RNA-seq data.^73^ DexSeq was used to analyse the differential exon usage across samples.^74^

### Quantification and statistical analysis

GraphPad PRISM 9.5.0 for windows was used to generate all graphs and perform statistical analysis. Results were presented as means±SD or median±interquartile range (25^th^ to 75^th^ percentile) as indicated. For comparison between groups of equal variance either unpaired t-test or one-way ANOVA were used depending on group size. If data were not normally distributed Kruskal-Wallis ANOVA or Welch’s t-test were used. Differences were considered significant at p<0.05. Details about statistical tests used for each experiment and the number of repeat experiments analysed (n) are provided in the figure legends.

## Data Availability

• The RNA-seq data for this study have been deposited at ArrayExpress (https://www.ebi.ac.uk/biostudies/ArrayExpress/studies/E-MTAB-12578) and are publicly available. The source data for main and supplemental figures in the paper are available at Mendeley Data. Accession number and DOI for the datasets are listed in the key resources table. • This paper does not report original code. • Any additional information required to reanalyse the data reported in this paper is available from the lead contact upon request.

## Supplemental information

Document S1. Figures S1–S7 and Tables S3 and S4

Table S1. PTBP1 deficiency severely alters the transcriptome of MCF10A cells, related to Figure 6 RNA was isolated from MCF10A EditR (wt) and PTBP1 clone 4.1 (PTBP1) and send to Novogene (UK) for RNA library preparation, sequencing and bioinformatic analysis. Table includes results for genes differentially expressed in PTBP1-deficient cells. The following information is included: column A: Gene ID; column B-G: read count values of each sample after normalization; column H-I: standardized average of read counts for each group of samples; column J: log2 fold change of gene expression level between the PTBP1 group and the control group; column K: P value in hypergeometric tests; column L: corrected p value of multiple hypothesis test; column M: gene name; column N: chromosome gene is located on; column O-R: starting and end position, strand information and gene length of gene on chromosome; column S-U: Gene type, gene description and transcription factor family information for the gene of interest; column V-Z: Raw read count values of each sample; column AB-AG: FPKM values of each sample. Table includes information from three independent experiments.

Table S2. List of genes associated with DNA replication, DNA damage repair, S-phase checkpoint signaling, S-phase progression, and G1-S phase transition obtained from Gene Ontology database, related to Figure 6
