# Supplementary material for: PTBP1 enforces ATR-CHK1 signaling determining the potency of CDC7 inhibitors
Source: iScience. 2023 May 26;26(6):106951. doi: 10.1016/j.isci.2023.106951 (PMC10291475; doi:10.1016/j.isci.2023.106951)
Supplement: Document S1. Figures S1–S7 and Tables S3 and S4 [file mmc1.pdf]

**iScience, Volume *26***

## Supplemental information

# PTBP1 enforces ATR-CHK1 signaling determining the potency of CDC7 inhibitors

**Anja Göder, Aisling Quinlan, Michael D. Rainey, Declan Bennett, Daniel Shamavu, Jacqueline Corso, and Corrado Santocanale**

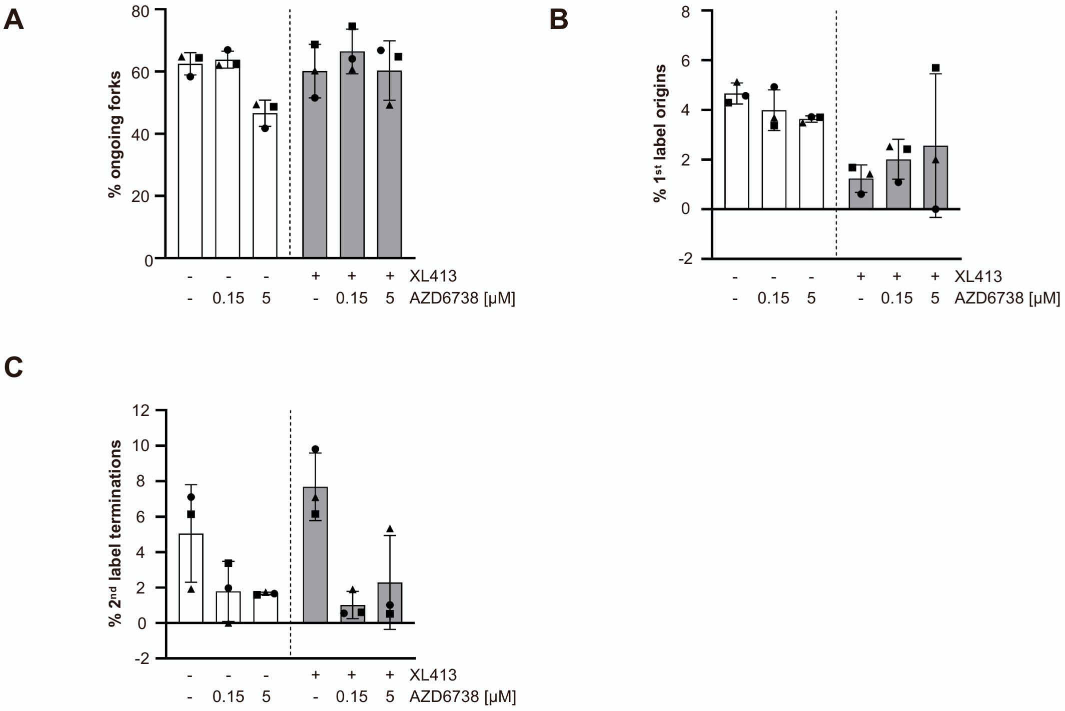


**Figure S1: Combination of ATRi and CDC7i has no significant effect on fork progression, related to Figure 1**.

**A.** MCF10A EditR cells were either treated with DMSO or 20 µM XL413 and labelled with CldU (cyan) for 30 min. CldU was washed off, AZD6738 (0.156 µM or 5 µM, as indicated) or DMSO was added and cells were labelled with IdU for 30 min in the continued presence or absence of XL413. Percentages of CldU-IdU (cyan-red) tracks representing ongoing forks are plotted as mean±SD. At least 150 replication tracks were analysed for each condition of three independent experiments.

**B**. MCF10A EditR cells were labelled as described in **A**. Percentages of IdU-CldU-IdU (red-cyan-red) tracks representing 1$^{st}$ label origins are plotted as mean±SD. At least 150 replication tracks were analysed for each condition of three independent experiments.

**C**. MCF10A EditR cells were labelled as described in **A**. Percentages of CldU-IdU-CldU (cyan-red-cyan) tracks representing 2$^{nd}$ label terminations are plotted as mean±SD. At least 150 replication tracks were analysed for each condition of three independent experiments.

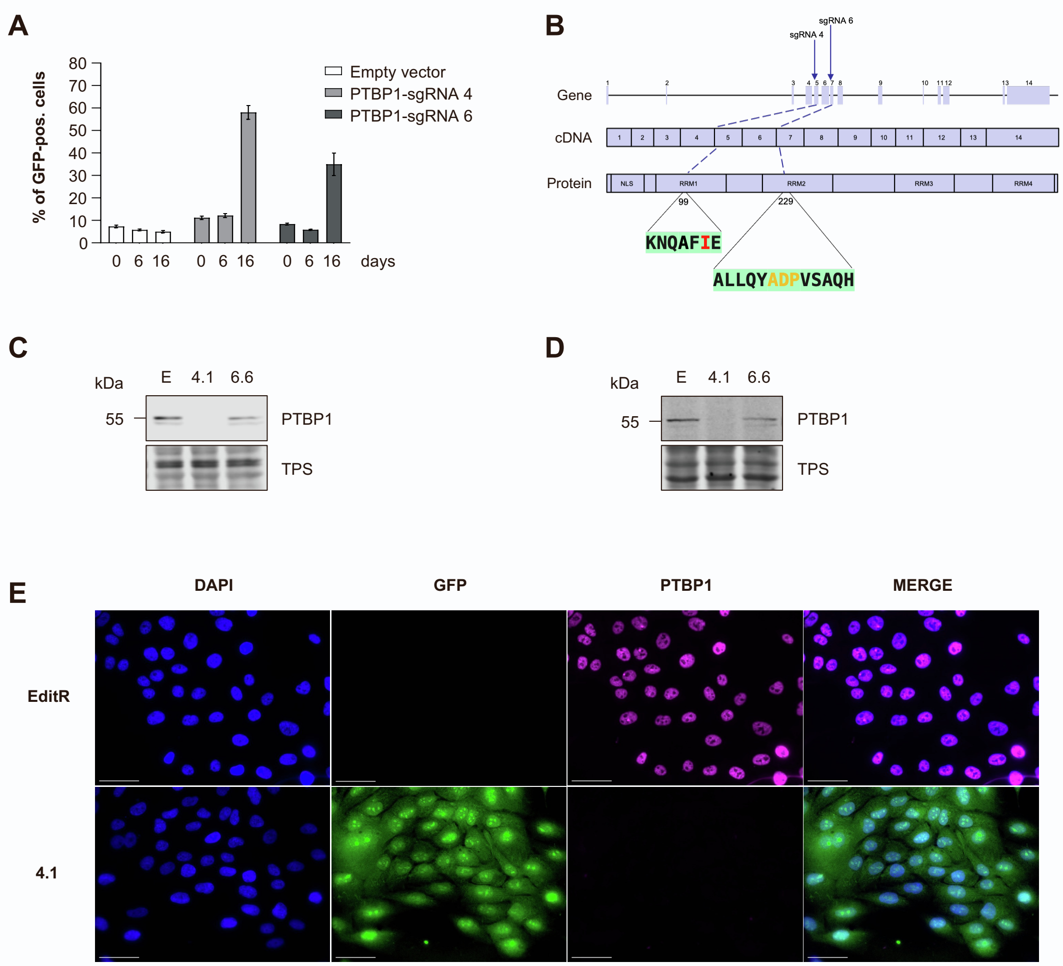


**Figure S2: Generation of PTBP1 mutant cell lines 4.1 and 6.6, related to Figure 2**.

**A.** MCF10A EditR cells were transduced at a low MOI with a lentiviral vector only expressing GFP or co-expressing GFP and PTBP1-sgRNA4 or PTBP1-sgRNA6. Cells were treated with 20 μM XL413 for 16 days. Percentage of GFP-positive cells in the population was measured at the indicated times by flow cytometry. The data shown represent three independent experiments, mean±SD.

**B.** Schematic showing sgRNA4 targeting exon 5 of PTBP1, which in MCF10A PTBP1 mutant clone 4.1 resulted in a homozygous loss of amino acid Ile99, positioned in RNA recognition motif 1 (RRM1). sgRNA6 targeted exon 7 of PTBP1, which in PTBP1 mutant clone 6.6 resulted in a heterozygous mutation, where one allele displayed a 9-nucleotide loss, resulting in the loss of 3 amino acids from position 229-231. Ile99 lost in clone 4.1 is marked in red, amino acids lost in clone 6.6 are in orange.

**C.-D** Whole cell extracts of MCF10A EditR (E), PTBP1 mutant clone 4.1 (4.1) and clone 6.6 (6.6) were prepared and analysed via immunoblotting. PTBP1 protein levels were detected using two different antibodies: sc-56701 (**C**) and sc-515282 (**D**).

**E.** Representative images of MCF10A EditR cells and PTBP1 mutant clone 4.1. Immunofluorescence used to detect PTBP1 (magenta), GFP (green), DAPI (blue). Images were collected on an IX71 Olympus microscope using a 60X oil-immersion objective lens (Scale bar, 50 μm). Brightness of images was adjusted as described in STAR Methods to aid visualisation.

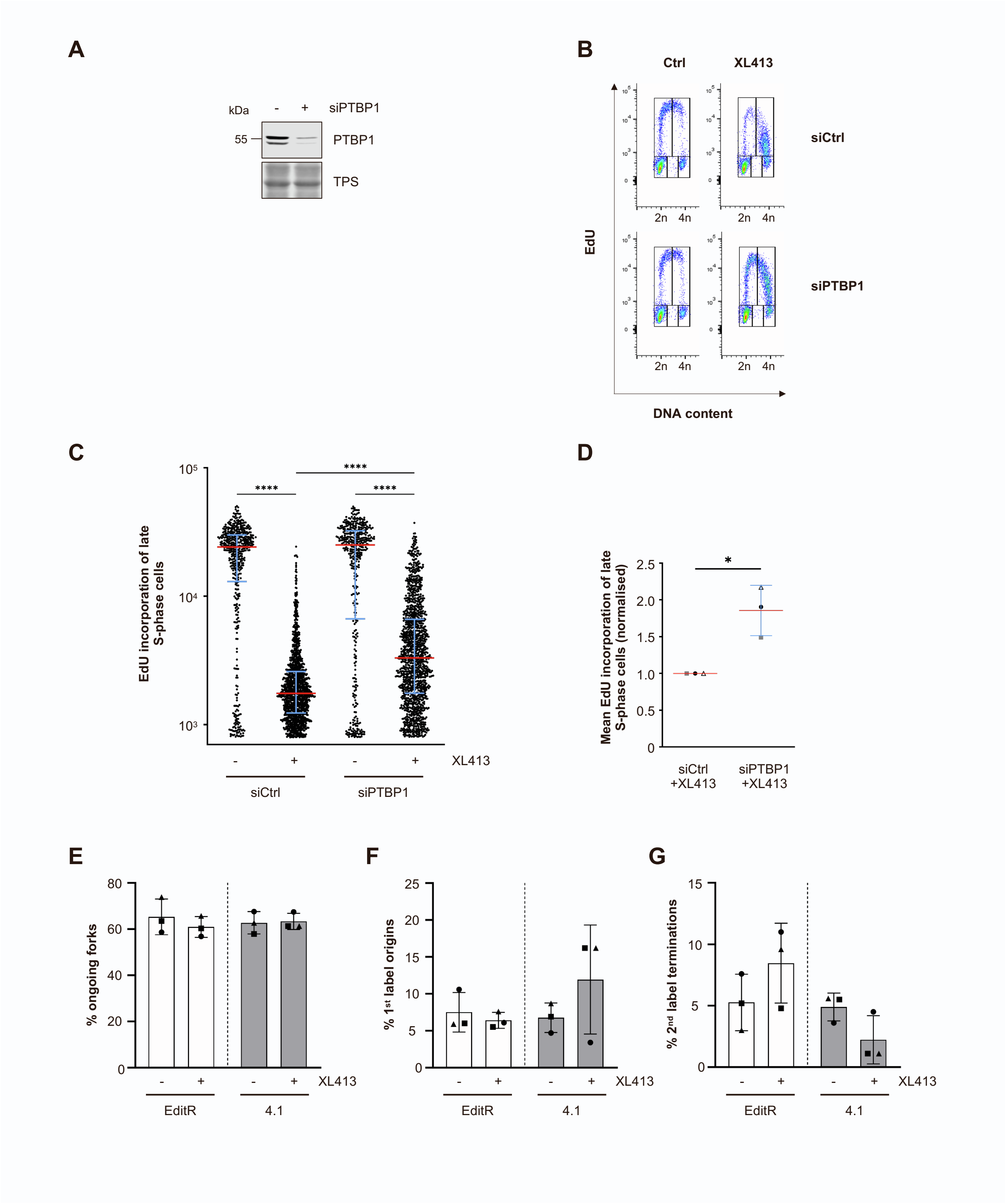


**Figure S3: PTBP1 deficiency restores efficient DNA synthesis in cells treated with XL413, related to Figure 3.**

**A.** MCF10A EditR cells were transfected with siRNA targeting either a non-protein control (siCtrl) or PTBP1 (siPTBP1) and incubated for 48 hours. Cells were then treated with 20 µM XL413 for 24 hours. Whole cell extracts were prepared and analysed by immunoblotting. Total protein staining (TPS) was used as loading control. Blots are representative of three independent experiments.

**B.** Cells were treated as described in **A**, labelled with 10 μM EdU 30 min prior to harvesting and analysed by flow cytometry. Representative dot blots of one of three independent experiments are shown.
**C.** Graphs showing fluorescence intensity, proportional to EdU incorporation, of individual cells in late S-phase for representative experiment described in **B**. Red lines indicate the median and blue lines show the interquartile range extending from the $25^{th}$ to the $75^{th}$ percentile of 448 cells. Statistical analysis was performed using one-way ANOVA, with Tukey's multiple comparison test (****p<0.0001).
**D.** Mean fluorescence intensity in late S-phase cells from three independent experiments expressed as a ratio relative to the XL413-treated siCtrl samples. Red and blue lines show the means±SDs. Statistical analysis was performed using Welch's t-test (*p < 0.05).
**E.** MCF10A EditR or PTBP1-deficient clone 4.1 cells were labelled with CldU (cyan) for 30 min. CldU was washed off and cells were labelled with IdU (red) for 30 min in the presence or absence of 20 μM XL413. Percentages of CldU-IdU (cyan-red) tracks representing ongoing forks are plotted as mean±SD. At least 150 replication tracks were analysed for each condition of three independent experiments.
**F**. MCF10A EditR or PTBP1-deficient clone 4.1 cells were labelled as described in **E**. Percentages of IdU-CldU-IdU (red-cyan-red) tracks representing $1^{st}$ label origins are plotted as mean±SD. At least 150 replication tracks were analysed for each condition of three independent experiments.
**G**. MCF10A EditR or PTBP1-deficient clone 4.1 cells were labelled as described in **E**. Percentages of CldU-IdU-CldU (cyan-red-cyan) tracks representing $2^{nd}$ label terminations are plotted as mean±SD. At least 150 replication tracks were analysed for each condition of three independent experiments.

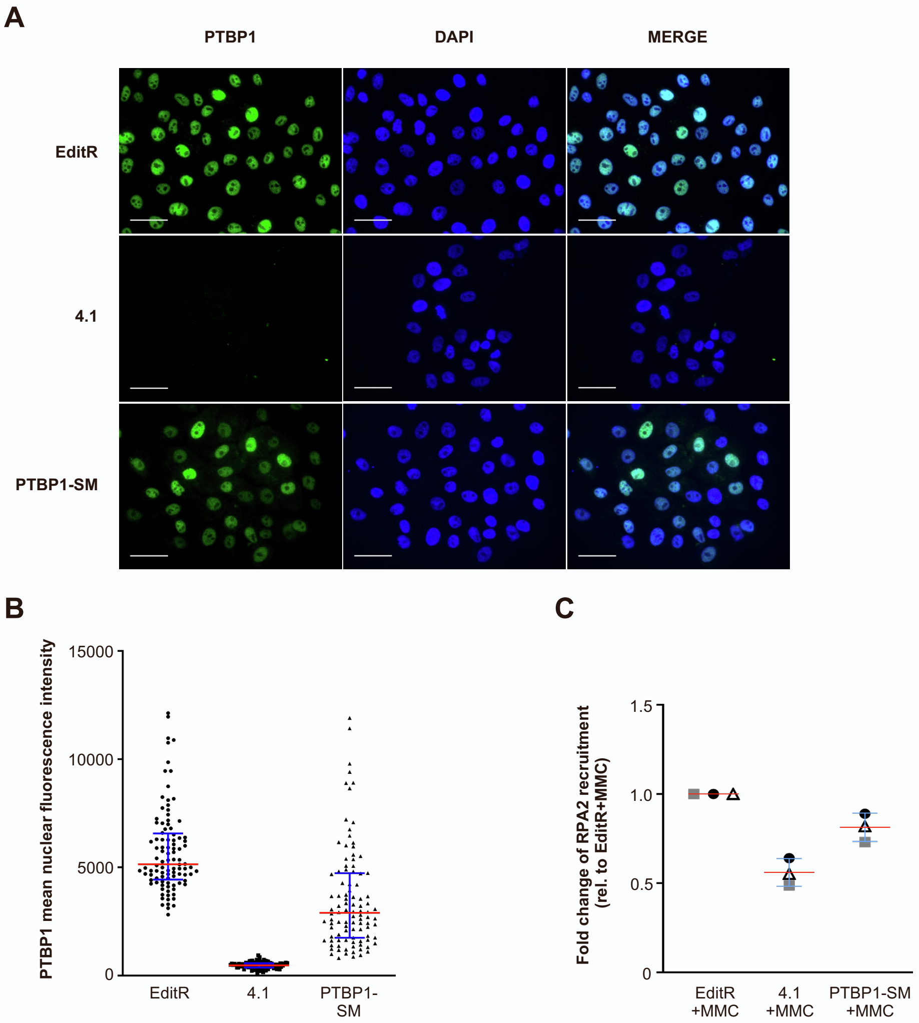


**Figure S4: Characterisation of MCF10A PTBP1-deficient clone 4.1 cells and clone 4.1 cells expressing PTBP1-SM allele, related to Figure 4.**

**A.** MCF10A EditR, PTBP1-deficient clone 4.1 and clone 4.1 expressing PTBP1-SM cells (PTBP1-SM) were fixed, immunostained with anti-PTBP1 antibody (green) and analysed by immunofluorescence. DAPI was used to visualise DNA (blue). Representative images of two independent experiments are shown (Scale bar, 50 µm). Brightness of images was adjusted for all samples to aid visualisation.

**B.** Mean nuclear fluorescence intensity of PTBP1 was manually determined using ImageJ software as described in STAR Methods. Graph represents fluorescence intensity of 100 nuclei in one of two independent experiments. The red lines indicate the median and blue lines show the interquartile range extending from the 25$^{th}$ to the 75$^{th}$ percentile.

**C.** MCF10A EditR, PTBP1-deficient clone 4.1 and PTBP1-SM cells were treated with 200 nM MMC for 24 hours. Chromatin-enriched fractions were prepared and analysed by immunoblotting with the indicated antibodies. Total protein staining (TPS) was used as loading control. Recruitment of RPA2 to the chromatin was quantified using Licor Empiria software. Signals were normalised to corresponding TPS and expressed relatively to MCF10A EditR+MMC samples. Graph represents quantification of three independent experiments one of which is displayed in main figure **4D**, mean±SD.

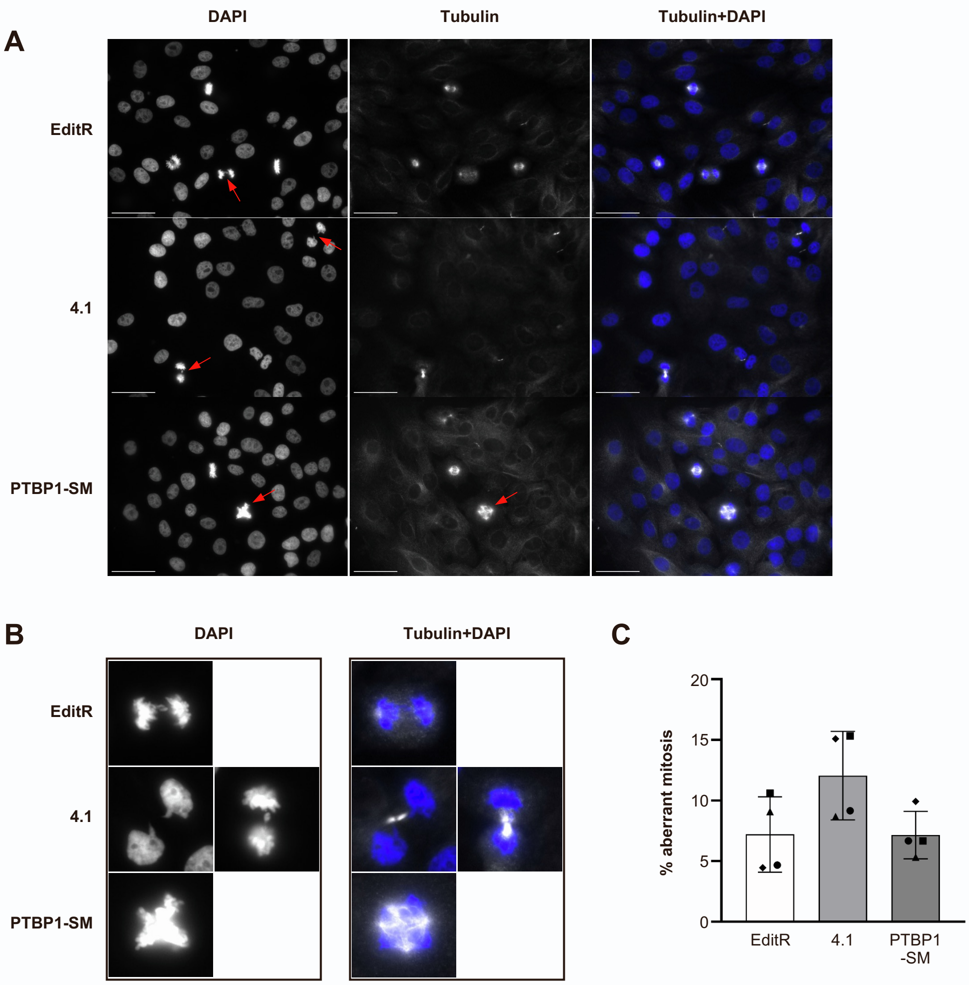


**Figure S5: PTBP1 deficiency increases the number of mitotic aberrations, related to Figure 5**

**A.** MCF10A EditR, PTBP1-deficient clone 4.1 and clone 4.1 expressing PTBP1-SM cells (PTBP1-SM) were fixed, immunostained with anti-beta-Tubulin antibody (grey, middle panel and right panel) and analysed by immunofluorescence. DAPI was used to visualise DNA (grey in left panel, blue in right panel). Representative images of four independent experiments are shown. Red arrows indicate cells with mitotic aberrations (as defined in STAR methods). Scale bar represents 50 µm. Brightness of images was adjusted for all samples to aid visualisation.

**B**. Cells with mitotic aberrations (red arrows) in representative images displayed in **A** were enlarged to improve visualisation.

**C**. Analysis of mitotic aberrations displayed in **A** and **B**. Graph shows percentage of mitotic cells with aberrations for four independent experiments, mean±SD. At least 150 mitotic cells were analysed per condition per experiment.

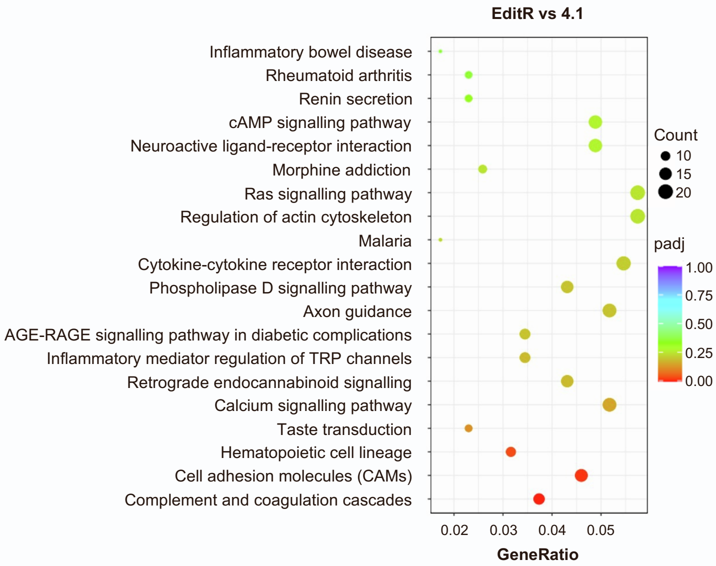


**Figure S6: PTBP1 deficiency results in altered gene expression, related to Figure 6.** Kyoto Encyclopaedia of Genes and Genomes (KEGG) analysis was performed to display pathways significantly altered in MCF10A EditR vs PTBP1 mutant 4.1 cells. The names of the 20 most enriched pathways are listed. The size of the circles indicates the number of genes corresponding to the pathway. The P adjusted value (padj) is visualised on a colour scale with red being the most significant.

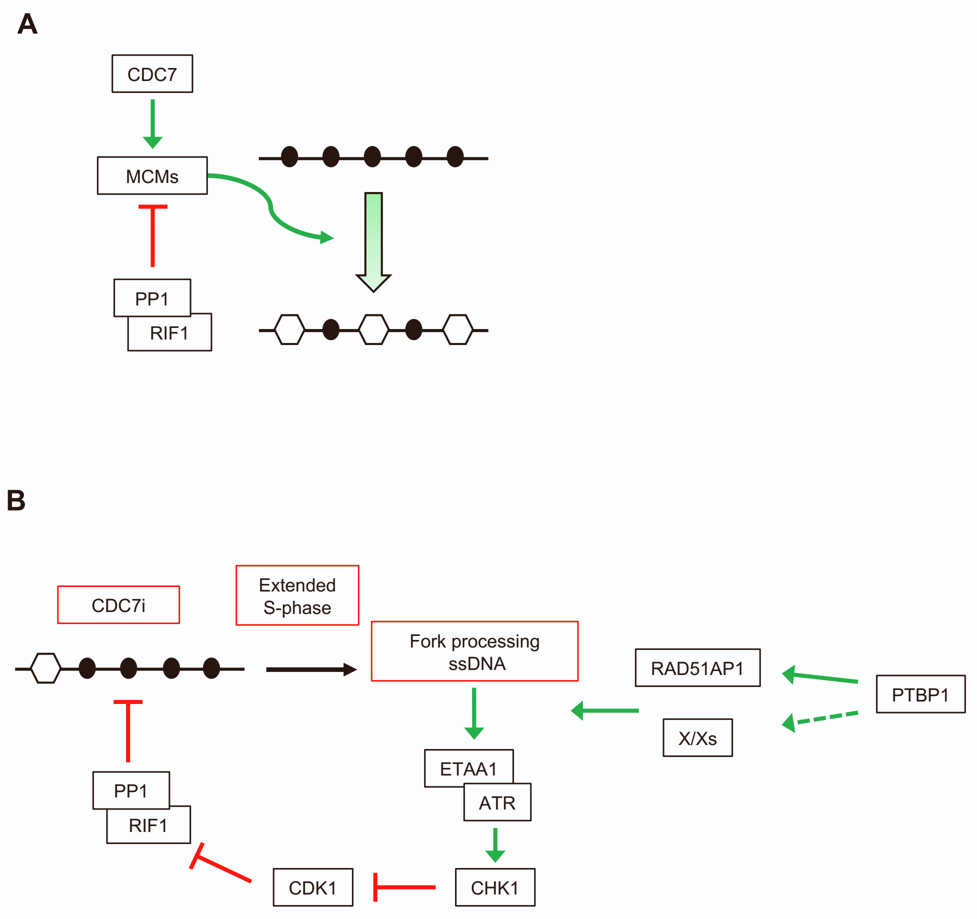


**Figure S7: Model of PTBP1's role in DNA replication and the replication stress response, related to DISCUSSION.**

**A.** CDC7-mediated phosphorylation of the MCM complex promotes origin activation during unperturbed S-phase. The RIF1-PP1 phosphatase complex counteracts this process. Black circles represent licensed replication origins, while open hexagons symbolise active replication forks.

**B.** Inhibiting CDC7 kinase reduces origin activation and requires active replication forks to travel longer distances, thereby extending S-phase. This delay in combination with ssDNA, created by replication forks encountering obstacles, activates the ETAA1-ATR-CHK1 pathway. Activation of the intra S-phase checkpoint inhibits CDK1, which leads to further inhibition of origin firing through the RIF1-PP1 phosphatase complex. The presence of PTBP1 allows for the efficient activation of the ATR checkpoint by the correct splicing of members of the HR and replication stress response like RAD51AP1 and likely other yet undiscovered factors (X/Xs). PTBP1 loss of function impairs ATR activation, thereby likely reducing the suppression of origin firing and altering the frequency of fork staling or processing. This defect in checkpoint signalling is accompanied by an increase in genomic instability.

| Gene name | PTBP1 DMSO_1 read count | PTBP1 DMSO_2 read count | PTBP1 DMSO_3 read count | WT DMSO_1 read count | WT DMSO_2 read count | WT DMSO_3 read count | PTBP1 DMSO | WT DMSO | log2 Fold Change | p value | padj |
|---|---|---|---|---|---|---|---|---|---|---|---|
| **MRN complex** | | | | | | | | | | | |
| ***MRE11*** | 1459.08942 | 1233.44546 | 1414.23372 | 2150.69097 | 1961.17266 | 2106.61212 | 1368.92287 | 2072.82525 | -0.5984953 | 2.0378E-09 | 3.4223E-08 |
| ***RAD50*** | 875.453651 | 643.450682 | 769.481305 | 378.399873 | 342.678019 | 670.737349 | 762.795212 | 463.938414 | 0.71687756 | 0.00121444 | 0.00562686 |
| ***NBN*** | 4346.55058 | 3997.31362 | 4199.52577 | 4232.39751 | 4164.85592 | 4218.19266 | 4181.12999 | 4205.1487 | -0.0082389 | 0.91229799 | 0.95443486 |
| **Fork protection** | | | | | | | | | | | |
| ***TIPIN*** | 1098.15677 | 1123.56388 | 1102.4115 | 1181.86556 | 1157.72451 | 1065.23028 | 1108.04405 | 1134.94011 | -0.0344925 | 0.71481951 | 0.83236329 |
| ***TIMELESS*** | 4220.80011 | 4262.61328 | 4343.44372 | 5198.17949 | 4919.80196 | 5375.83564 | 4275.61904 | 5164.6057 | -0.2725852 | 0.00028569 | 0.00161556 |
| ***CLSPN*** | 2308.62503 | 2063.00188 | 2247.99838 | 951.579306 | 764.435581 | 1541.20537 | 2206.54176 | 1085.74009 | 1.02298611 | 0.00112141 | 0.00527776 |
| **ATR/ATR activation** | | | | | | | | | | | |
| ***ATR*** | 2263.50845 | 2133.28649 | 2162.60706 | 2790.82588 | 2718.22749 | 2930.37695 | 2186.46733 | 2813.14344 | -0.3636288 | 6.9523E-06 | 5.8678E-05 |
| ***ATRIP*** | 1372.69597 | 1525.47308 | 1499.62504 | 1607.94584 | 1516.21844 | 1646.53598 | 1465.93136 | 1590.23342 | -0.1176126 | 0.20325038 | 0.35992547 |
| ***ETAA1*** | 665.229583 | 578.115689 | 625.563355 | 407.819703 | 370.092261 | 568.387798 | 622.969543 | 448.766587 | 0.47262029 | 0.00450895 | 0.0170341 |
| ***TOPBP1*** | 3328.06777 | 2979.67162 | 3289.00488 | 2654.88597 | 2412.45325 | 2955.21907 | 3198.91476 | 2674.1861 | 0.25837734 | 0.0106572 | 0.03489169 |

**Table S3: PTBP1 mutation results in an imbalance in the expression of protein complex subunits, related to Figure 6.** Differentially expressed genes in MCF10A PTBP1 clone 4.1 (PTBP1) and EditR (WT) cells were analysed for an imbalance in subunits of protein complexes involved in DNA replication, replication stress response and checkpoint signalling. Results for the MRN complex, fork protection complex as well as subunits involved in ATR activation are presented here. Table includes individual read counts for each sample of three independent experiments, the standardized average of read count for each group of samples (PTBP1 DMSO, WT DMSO), the log2 fold change of gene expression between PTBP1 mutant and control cells, p value as well as the adjusted p value (padj) of multiple hypothesis test.

**Table S4: Oligonucleotide sequences, related to STAR Methods.**

| Nucleotides | Sequence (5'-3') | Source |
|---|---|---|
| **Primers** | | |
| **PTBP1-sg4 fwd** | CTTCTGCAGCAAACGGAAATG | Merck |
| **PTBP1-sg4 rev** | CTCCTTGTGGTTGGAGAACTG | Merck |
| **PTBP1-sg6 fwd** | AGAACCTCTTCTACCCTGTGA | Merck |
| **PTBP1-sg6 rev** | GGCTCTTGTCATTGTTGTACTTG | Merck |
| **PTBP1-fwd** | AATCGGATCCGCGGCCGCATGGACGGCATTGTCCCAGATATAGC | Merck |
| **PTBP1-rev** | GATTGGATCCGCGGCCGCCTAGATGGTGGACTTGGAGAAGGAGAC | Merck |
| **RAD51AP1-fwd** | AATCGGATCCGCGGCCGCATGGTGCGGCCTGTGAGACATAAG | Merck |
| **Rad51AP1-rev** | GATTGGATCCGCGGCCGCTCAGGTGCTAGTGGCATTTGGATGC | Merck |
| **PTBP1-SDM fwd** | GTGTTCATCTCGATGAATGCCTGGTTTTTCCCCTT | Merck |
| **PTBP1-SDM rev** | AAGGGGAAAAACCAGGCATTCATCGAGATGAACAC | Merck |
| **sgRNA** | | |
| **sgRNA4** | CTCCGTGTTCATCTCGATGA | IDT |
| **sgRNA6** | CGGGGTCCGCATACTGCAGC | IDT |
| **siRNA** | | |
| **siCtrl** | GCAUAUCGUCGUAUACUAU | Merck |
| **siPTBP1** | CUGUGCCUAGCAAUAUU | Merck |
